# Determinants of Adoption, Implementation, Reach, and Sustainability of PrEP Services in a Sexual Health Clinic in Canada: A Qualitative Analysis Using CFIR and REAIM

**DOI:** 10.1177/23259582261458932

**Published:** 2026-06-11

**Authors:** Emma Nagy, Beatriz Alvarado, Miriam Kamotho, Nicole Szumlanski, Tianxiu Hugh Guan, Jorge Martinez-Cajas

**Affiliations:** 1 Knowledge Management, Southeast Public Health, Kingston, ON, Canada; 2 Department of Public Health Sciences, 4257 Queen's University, Kingston, ON, Canada; 3 Sexual Health Team, Southeast Public Health, Kingston, ON, Canada; 4 Division of Infectious Diseases, Department of Medicine, 4257 Queen's University, Kingston, ON, Canada

**Keywords:** HIV pre-exposure prophylaxis, implementation science, sexual health clinics, RE-AIM, CFIR

## Abstract

**Background:**

Sexual health clinics are promising settings for HIV pre-exposure prophylaxis (PrEP), yet implementation in mixed urban–rural regions remains underexplored. This study evaluated the implementation of a PrEP clinic in a mid-sized Ontario region within a public health setting, including adoption, implementation model, reach, perceived effectiveness, and key determinants.

**Methods:**

Guided by RE-AIM and the Consolidated Framework for Implementation Research (CFIR), we conducted an evaluation using process-mapping and interviews with staff (n = 7) and clients (n = 5).

**Results:**

Adoption and implementation were supported by strong leadership, organizational culture, provider knowledge, and perceptions of PrEP as effective and simple. The clinic demonstrated high fidelity, sustained service delivery, and meaningful client engagement. However, reach remained uneven, with the program primarily serving insured populations. Medication costs, poverty, stigma, and geographic barriers limited equitable access.

**Conclusions:**

Sexual health clinics can sustainably deliver PrEP, but equitable scale-up requires structural reforms, diversified delivery models, and stronger community partnerships.

## Plain Language Summary Factors that Helped a Canadian Sexual Health Clinic Start and Sustain HIV Prevention Services (PrEP)

HIV pre-exposure prophylaxis (PrEP) is a medication that can prevent HIV infection when taken regularly. While PrEP programs have expanded in large cities, less is known about how these services can be delivered in smaller regions that include rural communities. These areas often have fewer healthcare providers and fewer specialized services. Understanding how PrEP programs operate in these settings can help improve access to HIV prevention. This study evaluated a PrEP clinic located within a public health sexual health service in Southeastern Ontario, Canada. The goal was to understand how the clinic was developed, how it operates, what factors helped or challenged its implementation, and what lessons could help other regions offer similar services. Researchers collected information by mapping the clinic's workflow, interviewing clinic staff and clients. The clinic was created because community members wanted access to PrEP and there were few local providers able to prescribe it. Strong leadership, supportive staff, and clear clinical procedures helped integrate the program into existing sexual health services. During the COVID-19 pandemic, the clinic introduced telephone appointments alongside in-person visits. This hybrid model improved convenience for many clients and has continued to be used. Clients reported high satisfaction with the clinic and felt more confident managing their sexual health. No HIV infections occurred among clients receiving PrEP during the study period. However, most clients were gay or bisexual men with health insurance and experience navigating healthcare systems. Groups such as women, transgender people, and individuals facing financial or housing challenges were less represented. Sexual health clinics can successfully provide PrEP in mid-sized regions, but improving equitable access will require better funding, community partnerships, and outreach to underserved populations.

## Introduction

HIV pre-exposure prophylaxis (PrEP) has transformed HIV prevention, offering a highly effective strategy to reduce the risk of HIV acquisition.^1^ Scaling equitable access and uptake among populations at increased risk is critical to achieving the Joint United Nations Programme on HIV/AIDS (UNAIDS) goal of ending AIDS as a public health threat by 2030. PrEP use has increased across Canada since its approval in 2016 alongside declines in HIV diagnoses in high-coverage urban centers.^2^ However, uptake remains uneven across provinces and populations due to differences in drug coverage, provider engagement, and service delivery models.^3‐5^ In Ontario, uptake has been concentrated in large urban centers, with lower use in Northern and Eastern regions.^6,7^ Lower uptake outside major urban settings has been linked to provider perceptions of low HIV risk, limited organizational capacity, and challenges integrating PrEP into existing services.^8‐11^ Disparities in access persist among racialized populations, cisgender women, immigrants, and individuals living in rural areas.^12‐16^ Addressing these inequities will require delivery models capable of reaching diverse populations across geographic and service contexts. This is an opportune time to make such advancements, given recent updates to PrEP guidelines and expanded delivery options.^17^

Public health sexual health clinics have emerged as an important alternative delivery setting, given their mandate to provide accessible, equity-oriented sexually transmitted, and blood-borne infections prevention services. Several public health units (PHUs) in Ontario have implemented in-house PrEP services using a range of models,^18‐20^ which demonstrated effectiveness in terms of reduction of new HIV cases.^21^ However, most established models are in large urban centers, highlighting the need for adaptable and resource-efficient approaches for mid-sized and mixed urban–rural regions. A recent situational analysis of 12 public health sexual health clinics across mid-sized and mixed urban regions in Ontario found that only two offered on-site PrEP services with most of them relying of referral to external providers with not tracking of the engagement of clients to PrEP.^19,22^

Using the consolidated framework for implementation research and equity lens, the comparison across the 12 public health sexual health clinics^19^ suggested that adoption, implementation, and sustainment of PrEP in these settings presents challenges that can only be fully understood from a multilevel perspective. Common facilitators included strong belief in PrEP as an effective HIV prevention strategy, inner setting aspects, such as supportive organizational cultures, sexual health expertise, partnerships with community organizations and external providers, and alignment with public health mandates.^19^ Major barriers included COVID-19 recovery constraints, limited staffing and prescribing capacity, lack of public funding for PrEP medication and monitoring, limited access to primary care, uncertainty about workflow integration, and insufficient resources for outreach and navigation. The equity analysis showed that due to those structural factors, current PrEP delivery often reaches gbMSM but is less effective in reaching other equity-deserving populations, including people who use drugs, Indigenous communities, people experiencing homelessness, newcomers, uninsured clients, and rural residents.^19^

To better understand how PrEP can be successfully implemented in mid-sized and geographically diverse settings, we conducted an in-depth evaluation of one of those 12 public health sexual health clinic serving an urban, suburban, and large rural region. This clinic was selected because its model provides a pragmatic example of onsite PrEP delivery within a mixed regional context. It was also the only clinic among those examined that provided PrEP onsite, despite facing implementation challenges like those reported by other public health sexual health clinics. The present study is part of a broader evaluation conducted by our team at this clinic that specifically examines:

1. What factors motivated the initial adoption of a PrEP clinic in this mid-sized Ontario region?
2. What is the current clinic model and operational workflow? How and why was this model developed and adapted overtime? What were the key facilitators and barriers during early implementation, and which factors contribute to ongoing sustainment?
3. What is the reach and effectiveness of the PrEP clinic? What factors are associated with the clinic's reach, client engagement, and effectiveness outcomes? and
4. What actionable recommendations can inform other public health units or primary care settings seeking to integrate PrEP services?

## Methods

### Evaluation Context

The evaluation of the PrEP clinic was initiated by Southeast Public Health (formerly, KFL&A Public Health). At the time of this study, KFL&A Public Health has since merged with its two neighboring health unit regions and is now Southeast Public Health. The PrEP clinic located at the Kingston office was established in 2018 serving over 205,000 residents across a suburban-rural area in Southeastern Ontario. The PrEP clinic is part of the sexual health program comprised of an interdisciplinary team, including nurses, physicians, data clerks, and learners.

### Parent Evaluation Study

The evaluation of the experience of the “PrEP clinic” was part of a broader research project, an Endgame Grant, “Breaking New Ground,” by OHTN (EFP-1128-BNG) that aimed to more fully understand and investigate the PrEP landscape in primary care and public health units in Ontario (www.prepimplementationcollective.ca). The evaluation integrated qualitative and quantitative data sources to assess implementation processes, service reach, client engagement, and operational performance. A convergent mixed-methods approach was used to enable triangulation across data sources and generate a comprehensive understanding of implementation facilitators, barriers, and recommendations. The evaluation included four complementary components, each contributing to the assessment of the RE-AIM domains: (1) a process-mapping workshop, (2) leadership and staff interviews, (3) client interviews and surveys (structured surveys captured demographic characteristics, service utilization, fidelity indicators, and satisfaction measures), and (4) quantitative analysis of clinic administrative records. Data collection began in January 2024 with the quantitative analysis of administrative records covering the period from 2018 to 2024. Process mapping was initiated in May 2024, followed by leadership and staff interviews conducted between August and October 2024. Client surveys and interviews were conducted between September and October 2024.

The analysis of clinic records from 2018 to 2024 provides evidence of both reach and effectiveness.^23^ During this period, the clinic invited 141 clients to consider PrEP, and 70% of those who expressed interest initiated it. Client profiles indicate that the clinic primarily reached gay, bisexual, and other men who have sex with men at elevated risk of HIV acquisition, as reflected by 27% having a history of a sexually transmitted infection and 59% reporting condomless anal sex. No HIV seroconversions were observed during the evaluation period, and loss to follow-up was low at 7.6%. Although overall program disengagement was moderate at 53%, many clients remained adherent while actively engaged in care. Quantitative analysis of clinic records showed a median engagement duration of 8 months (IQR: 3-21), with approximately half of clients remaining on PrEP for at least 6 months, suggesting moderate to strong retention within the program.^23^

### Evaluation Framework

**Figure 1.**
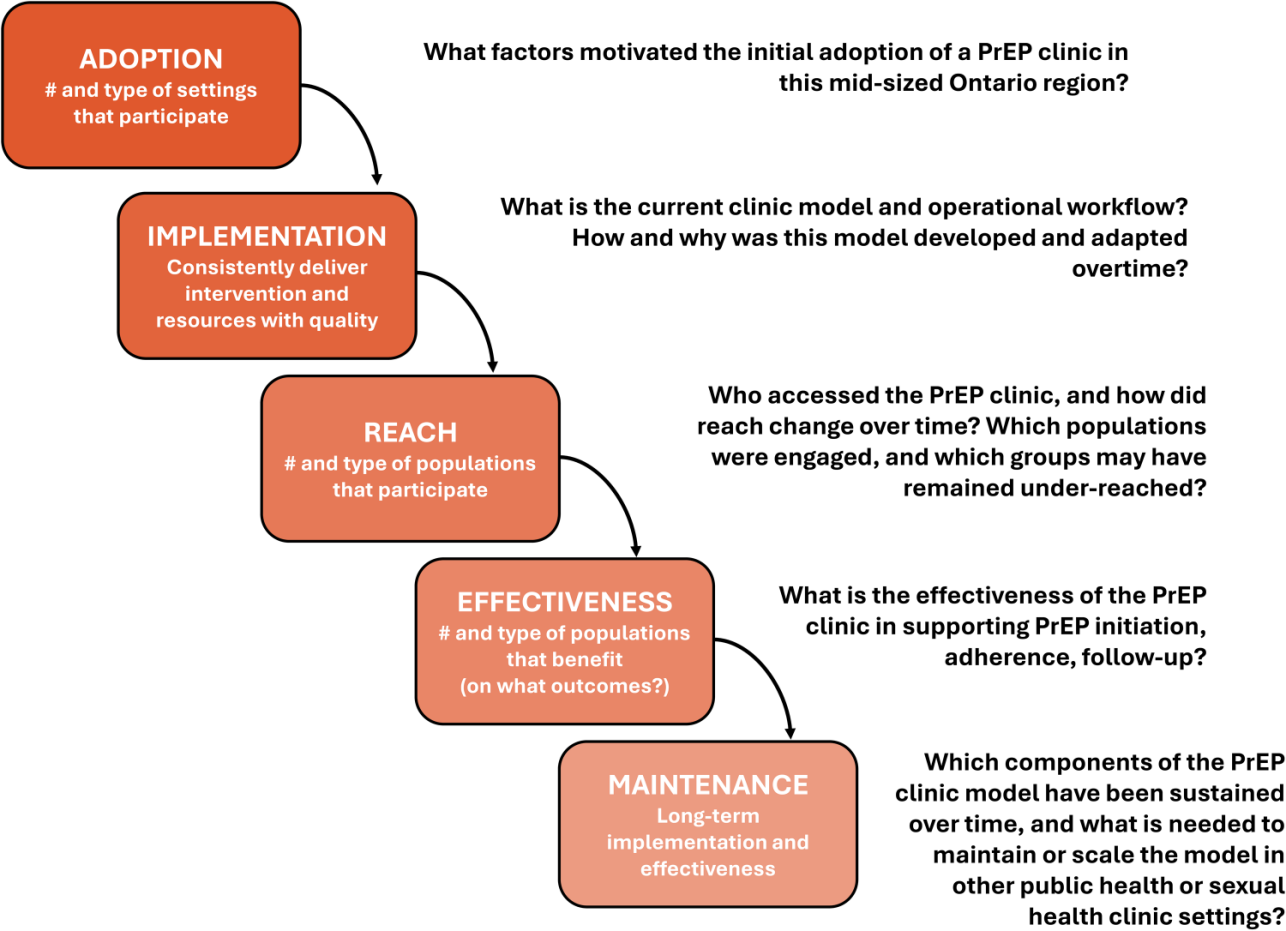
RE-AIM cascade use in this study.

To examine how and why the PrEP clinic model functioned in practice, we used RE-AIM^24^ to structure the evaluation of implementation outcomes and the CFIR^25^ to explain how and why those outcomes were achieved. The RE-AIM cascade describes how implementation outcomes are connected across sequential stages of a program's adoption, implementation, and sustainment (Figure 1). In this evaluation, the cascade begins with “adoption,” examining why and how the clinic decided to implement onsite PrEP. It then moves to “implementation,” describing how the model operates in practice, including fidelity to core components, acceptability, appropriateness, and adaptations over time (in Supplemental Table 1). These adoption and implementation processes, in turn, shape “reach,” or which populations are able to access and engage with the clinic, as well as who remains underserved. Reach then influences “effectiveness,” including biomedical outcomes, adherence, retention, efficiency, safety, and equity (Supplemental Table 2). Finally, the cascade ends with “maintenance,” which examines whether the model has become embedded in routine practice and what factors support or threaten its long-term sustainability.

**Figure 2.**
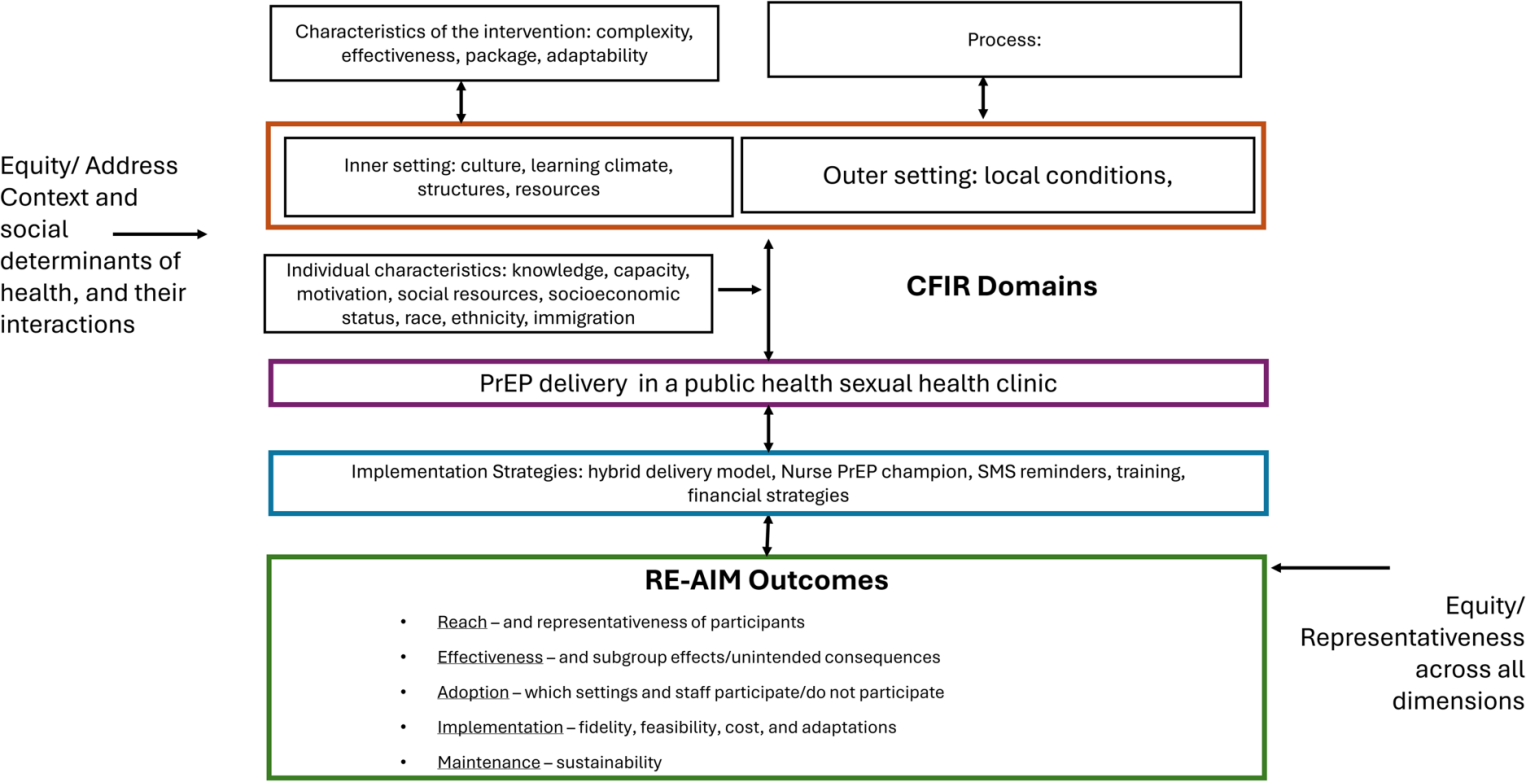
CFIR and its influence on RE-AIM (adapted from https://re-aim.org).

Following our previous work,^19^ we used CFIR to identify and interpret the contextual determinants influencing each stage of the RE-AIM cascade (Figure 2; Supplemental Table 3). CFIR has been widely applied in studies examining the adoption and implementation of PrEP services across public health and sexual health clinic settings.^26‐28^ The combined use of CFIR as a determinant framework and RE-AIM as an evaluation framework strengthens understanding of both implementation outcomes and the multilevel contextual factors that shape them.^29^ Although combining determinant and evaluation frameworks is strongly recommended in implementation science, and existent models are well known, this approach remains less commonly applied in the field of PrEP delivery.

Lastly, building on our previous work using CFIR and the Health Equity Implementation Framework (HEIF),^30^ we applied a health equity lens to examine how structural, organizational, and individual-level determinants shaped the implementation of the clinic model (Figure 2). These determinants included factors related to clients, clinic staff, external primary care providers, and the broader health system, all of which are captured across CFIR and HEIF domains. This lens helped us interpret our RE-AIM findings, particularly those related to reach, access, engagement, and sustained participation across the PrEP care cascade. It also allowed us to assess whether the current clinic model reduced, maintained, or reproduced inequities from PrEP access and initiation through adherence and follow-up care. By integrating RE-AIM outcomes with CFIR and HEIF determinants, the analysis generated actionable recommendations for adapting and integrating PrEP services into other public health and sexual health clinic settings. Table 1 presents the research questions aligned with the RE-AIM, CFIR, and HEIF frameworks.

**Table 1.** Objective, Research Question, and the Use of RE-AIM and CFIR.

RE-AIM Domain	Evaluation Question	CFIR	Equity-Oriented Questions	Actionable Recommendations
Adoption: The intention, initial decision, or action to try or employ an innovation or evidence-based practice—in this case PrEP delivery on-site	What factors motivated the initial adoption of a PrEP clinic in this mid-sized Ontario region?	What structural, organizational, intervention or individual factors motivated the initial adoption of the PrEP clinic?	Which factors have enabled or limited equitable adoption of the PrEP clinic?	What adaptations might be needed to facilitate adoption in other settings?
Implementation: At the setting level, implementation refers to fidelity to the various elements of an intervention's protocol, including consistency of delivery as intended and the time and cost of the intervention. At the individual level, implementation refers to clients’ use of the intervention strategies.	What is the current clinic model and operational workflow? How and why was this model developed and adapted overtime?	What were the key facilitators and barriers during early implementation, and which factors contribute to ongoing sustainment?	Do adaptations reduce or exacerbate health inequities over time? How do social determinants of health shape implementation and sustainability of the PrEP clinic over time?	Which core components of the model should be preserved, and which components can be adapted to local contexts? What strategies are needed to strengthen fidelity, acceptance, and appropriateness and cost associated to its implementation?
Reach The absolute number, proportion, and representativeness of individuals who are participating in the program	Who accessed the PrEP clinic, and how did reach change over time? Which populations were engaged, and which groups may have remained under-reached?	What factors are associated with the clinic's reach?	Are all populations equitably reached by the The PrEP clinic? Who is not reached by the PrEP clinic (in terms of a range of social dimensions and social determinants of health) and why?	How can we ensure that the clinic is reaching those who experience inequities related to social dimensions and social/structural determinants of health?
Effectiveness The impact of PrEP delivery on important outcomes, including potential negative effects, quality of life, economic outcomes, efficiency, etc.	What is the effectiveness of the PrEP clinic in supporting PrEP initiation, adherence, follow-up?	What factors shaped effectiveness and engagement?	Are PrEP benefits experience across all clients groups?	How can the clinic continue to provide a flexible, safe, and effective services to their clients?
Maintenance: The extent to which a program or policy becomes institutionalized or part of the routine organizational practices and policies. At the individual level, maintenance has been defined as the long-term effects of a program on outcomes after 6 or more months after the most recent intervention contact.	Which components of the PrEP clinic model have been sustained over time, and what is needed to maintain or scale the model in other public health or sexual health clinic settings?	What factors shaped long-term maintenance and sustainability?	Is the model being sustained equitably? Which populations continue to receive benefits over time, and which may be lost across the PrEP cascade? Are the determinants of sustainability the same across low-resource and high-resource settings?	What sustainability strategies are needed to maintain program over-time? Which components need to be adapted or changed to ensure sustainability?

### Current Work

The present analysis focuses primarily on qualitative findings coming from the 1) a process mapping workshop, (2) leadership and staff interviews; and (3) client interviews.

#### Process Mapping With the PrEP Clinic Team

An in-person session was facilitated by a research associate from the health unit (EN), and adjunct professor (BA), to map each step of the clinic's workflows at a high level. All staff of the “PrEP clinic” were eligible to participate. Participants included staff with expertise across all key clinic functions, including management, clinical service delivery, and administrative support. Using a structured facilitation guide (Supplemental Table 4), participants collaboratively mapped clinic workflows from referral and intake through follow-up and retention. The session aimed to identify decision points, bottlenecks, inefficiencies, strengths, and opportunities for optimization. Field notes and the resulting process map were documented, use to guide the other semistructured interviews and incorporated into the qualitative analysis. The finalized process map was created using draw.io and it is available for reference at: https://www.prepimplementationcollective.ca/evaluation-of-services.

#### Interviews With Leadership and Staff

Staff and leadership involved at different stages of the clinic's development—including its inception, key periods of change, and current operations—were purposively sampled based on their roles in the clinic's implementation and evolution and invited to participate in in-depth individual interviews. This approach aimed to capture a comprehensive understanding of facilitators and barriers to initial adoption, current operational processes, and perceived strengths, challenges, and risks associated with the clinic model. Interviews were audio-recorded with participant consent, transcribed using transcription software, and reviewed for accuracy by a member of the research team (EN). Interview guide was informed by RE-AIM and CFIR domains (Supplemental Table 5).

#### Interviews and Surveys With Clinic Clients

All current and former clinic clients were eligible to the study and were invited via email or text message to complete a short questionnaire and participate in an interview. Three rounds of invitations were sent. Participants therefore represent a self-selected convenience sample of clients who responded to the invitation. All participants provided informed consent prior to participation. Client interviews were conducted by a member of the research team (EN) via telephone or videoconference. Interviews explored client experiences with the clinic guided by RE-AIM (Supplemental Table 6).

#### Qualitative Data Analysis

Interviews were transcribed verbatim using the automatic transcription feature in Microsoft Teams and analyzed using deductive qualitative content analysis, consistent with the approach described by Hsieh and Shannon.^31^ The initial phase of analysis focused on developing a detailed process map of the PrEP Clinic model. This map was constructed using workshop discussions, field notes, and follow-up interviews with staff, allowing us to identify key operational steps, decision points, and role responsibilities. Subsequently, the RE-AIM framework was applied as a set of predefined categories to guide data organization and coding across all interviews, including both staff and clients. The research questions and operational definitions presented in Table 1 and Supplemental Material informed the development of a structured coding framework within NVivo software. One researcher (BEA) independently coded all transcripts according to the RE-AIM domains and systematically identified themes within each dimension and subdimension. Then, a second researcher (EN) reviewed coded transcripts and the emerging thematic structure to enhance analytic rigor and consistency. Following RE-AIM coding, an initial analytic summary was developed for each domain to synthesize the main findings. This first analytic output was used to prepare a report, which was independently reviewed by three staff members to assess accuracy, ensure alignment with their experiences, and provide recommendations for additional analysis.

CFIR was then incorporated to support a deeper contextual interpretation of the findings. This involved generating additional codes nested within the existing RE-AIM categories to capture more explicitly the determinants shaping each RE-AIM domain. This phase of coding was conducted in NVivo by a researcher with extensive experience applying CFIR (BEA). The CFIR codes nested within the initial RE-AIM domains were exported to Excel for interpretation, and an initial RE-AIM–CFIR analytic report was prepared, including the main findings and supporting narratives. An additional round of interpretation was then conducted with a second researcher (EN) to validate the coding and interpretation. Factors associated with each RE-AIM domain were synthesized as facilitators or barriers, with attention to their implications for overall implementation and equitable PrEP delivery.

To enhance credibility and trustworthiness, clinic staff participated in two focus group discussions to review and validate preliminary findings, assess alignment with their experiences and organizational context, and refine interpretation of implementation barriers, facilitators, and recommendations. Although a comparable member-checking process was not feasible with client participants, the analysis incorporated multiple strategies to strengthen rigor, including triangulation across qualitative interviews (clients and staff), process mapping, client surveys, and clinic administrative records; use of established implementation frameworks; maintenance of an audit trail documenting coding and analytic decisions; and iterative review of findings by staff and researchers.

### Reflexivity

Reflexivity was incorporated throughout the evaluation with the recognition that the research team's experience and beliefs about PrEP could not be fully separated from the way the study was designed, conducted, and interpreted. The team included investigators with backgrounds in HIV prevention, public health, implementation science, and qualitative research; one investigator worked in public health but was external to the clinic. These experiences supported a strong contextual understanding of PrEP delivery, but may also have shaped how the intervention was perceived, including assumptions about its value, relevance, and potential impact. Rather than claiming complete neutrality, the evaluation remains closely grounded in participants’ narratives, including both positive and critical accounts of the clinic model.

## Results

### Participants

Seven key informants were interviewed from the PrEP clinic in this study, which represented a majority of the clinic's nine staff members at the time. Participants represented management, primary care providers, nursing staff, and administrative personnel. A total of 102 clients were invited, of which 20 completed the survey, of whom five accepted to be interviewed. Clients surveyed and interviewed included a mix of current and former clinic clients. Given the small number of participants, no demographic aspects of the sample of staff or clients are provided.

### Adoption

Across interviews, staff described PrEP as an effective, evidence-based, and necessary HIV prevention intervention, particularly in the absence of an HIV vaccine or cure.

“I personally think it's valuable just because it is one of these interventions that there has been research that shows that this intervention does decrease, you know, HIV infections […] We're never gonna get a HIV vaccine anytime soon and we're not gonna get a HIV cure any time soon.”

This strong evidence base reinforced the legitimacy of PrEP within the public health unit and aligned with broader public health priorities, including reducing HIV incidence and improving access for equity-deserving populations. As one staff member noted, PrEP was viewed as valuable both for clients and as a learning opportunity for trainees rotating through the agency: I do see us holding the HIV Prep clinic also valuable from the education standpoint for all of the medical students, residents, nursing students even that come through the agency. Seeing HIV Prep is valuable.

Staff also described PrEP care as relatively simple to integrate into existing clinical practice once workflows and processes were established. One staff member explained: “This is way easier than dealing with high blood pressure. Some of the easiest type visits biomedically to do.” This perception of low complexity helped reduce initial hesitation and contributed to internal acceptance of the service. Although adoption initially required adjustments to workflows, electronic systems, and financial processes, staff generally found implementation more straightforward than anticipated. As one staff summarized: “Overall the feedback was like, Oh yeah, this is very straightforward. […]. Because there is some hesitancy right, about thinking it's like a big complicated thing to add. So yeah, very straightforward.”

PrEP delivery was also seen as cost-effective. Staff noted that shifting away from external physicians and after-hours nursing reduced financial strain: “For us it was actually cost saving because we shifted away from time and a half nursing time as well as paying an external physician.” Prior to adopting the PrEP clinic model, the organization operated three evening sexual health clinics per week, which staff described as financially unsustainable and difficult to staff with both nurses and physicians. Leadership identified an opportunity to reorient one of these clinics into a dedicated PrEP service offered during regular business hours.

Outer-setting factors also influenced adoption. Staff described increasing community demand and limited regional access to PrEP prescribers, particularly for individuals without a family physician. One staff recalled: “We had various phone calls actually for people calling in about HIV PrEP. They’d be like, where do I access it?”. At the time, PrEP access in the region was limited, with few prescribing options outside the infectious disease clinic, which often required referrals. This created an important access barrier for clients without a regular primary care provider and an opportunity for the health unit to incorporate a PrEP clinic into its services. As one staff member explained, “the clinic continued to provide sexual health services, but in a way that was more targeted to equity-deserving population[s] while also decreasing the resource needs of one clinic.” Staff described support from unit directors and clinic managers as essential to developing the clinic and protecting its continuity over time. This support helped create an implementation climate in which PrEP was viewed as compatible with the clinic's mandate and workflow, even in the absence of new dedicated funding.

Early adoption also required staff preparation and learning. Staff completed training modules and reviewed supporting materials on PrEP provision; however, participants emphasized that training needs extended beyond clinical knowledge. Staff also needed to understand how PrEP would function operationally within the clinic, including history-taking, scheduling, laboratory processes, documentation, and financial workflows. As one staff member explained: “resources existed for learning about PrEP itself, but the more important challenge was understanding the implementation of PrEP and how does it fit in your own organization … all the nitty gritty logistics.”

### Clinic Model and Its Implementation

#### Clinic Model

**Figure 3.**
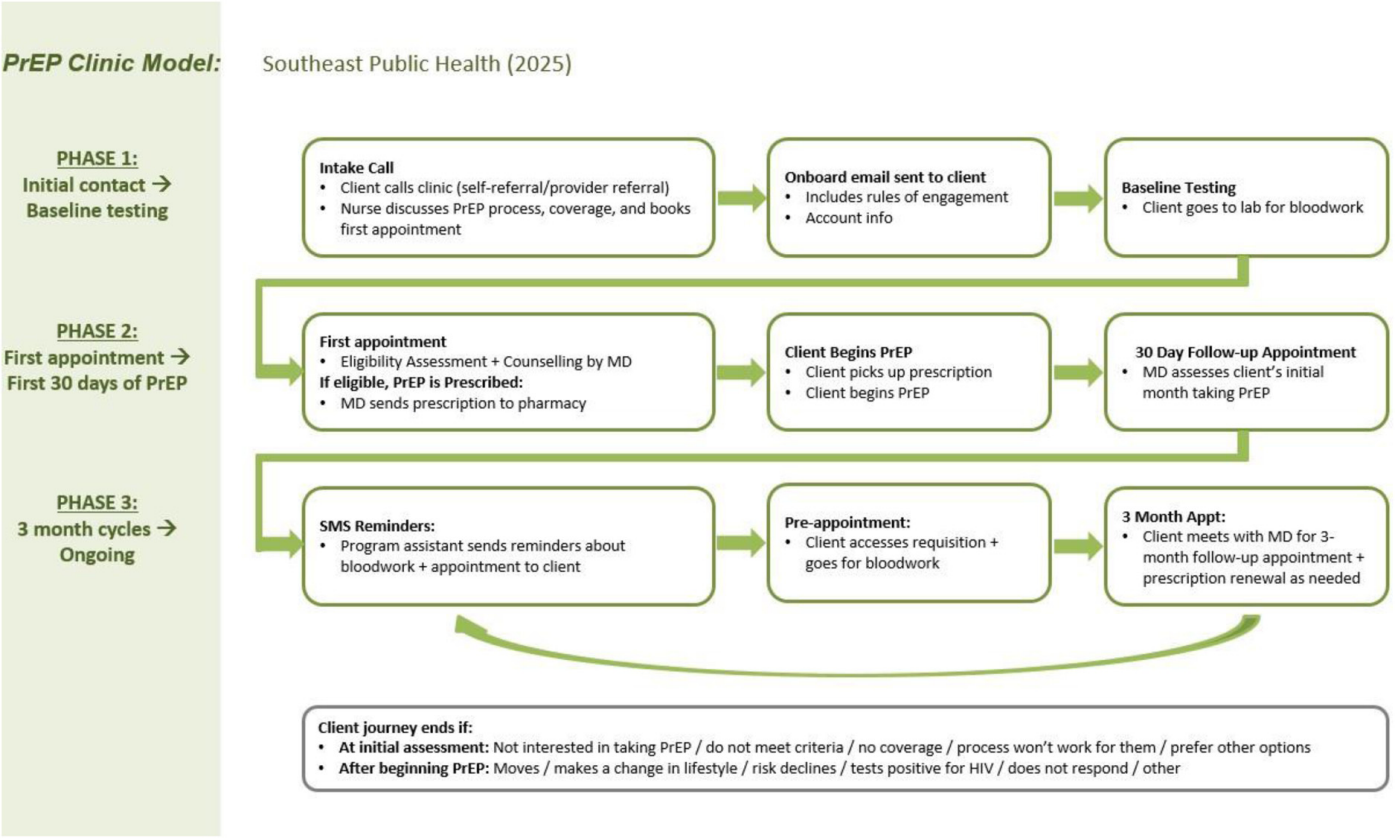
PrEP clinic process map—southeast public health (2025 clinic model).

Since November 2020, the PrEP Clinic has operated a mixed phone and in-person care model that supports access through external provider referrals, self-referrals, and referrals from sexual health clinic staff (Figure 3). The process begins with appointment scheduling and confirmation by phone or SMS, followed by an initial consultation, usually by phone, in which nursing staff provide counseling and assess eligibility using the HIRI-MSM tool together with clinical judgment.

Eligible clients complete baseline laboratory testing using online requisitions and, if results are normal, meet with a physician for further counseling and a PrEP prescription. Follow-up care occurs within 30 days and then every 3 months, primarily by phone, with at least one annual in-person visit. Clients complete required STBBI and kidney function testing before each follow-up, usually through local laboratories, and receive ongoing counseling on medication use, side effects, adherence, vaccination, and funding options.

Care is documented in the OSCAR electronic medical record system, with nurses coordinating clinical follow-up and support, a nurse lead overseeing protocols and onboarding, physicians prescribing medication every 3 months, and program assistants managing scheduling, reminders, and OHIP billing.

#### Fidelity

Staff and clients consistently reported that key service elements were delivered as intended, including standardized educational materials, structured PrEP counseling, risk assessment and decision support, routine laboratory monitoring, vaccination review, and facilitation of medication access through external pharmacies. Staff emphasized that visits included both biomedical monitoring and broader preventive care, including vaccination discussions, site-specific testing, and other sexual health needs: “We make sure we have a conversation with them about additional vaccines … and offering site-specific testing.”

Clients similarly reported receiving clear explanations about the rationale for quarterly monitoring, including kidney function and STBBI testing. One client explained that staff “answered any questions that I had” and “explained the reasoning for the … every 3 month testing, checking the kidneys, that kind of stuff.”

Staff also described using a structured intake and risk assessment process across different access points, including telephone and in-person encounters. As one staff member explained, “whether that's in … the quick test clinic or on the phone—the sexual health line—then what we do is we just have a brief sort of intake process, after which clients are assessed using the HIRI scoring tool.”

#### Adaptations

The most significant adaptation occurred during the COVID-19 pandemic, when the clinic shifted from primarily in-person care to telephone-based visits. Over time, this adaptation evolved into the hybrid model (three telephone visits and one in-person visit annually). Both clients and staff described this model as convenient, acceptable, and easier to navigate. One client reflected that “COVID really threw a wrench into getting blood work done,” but that the shift to phone visits became “super convenient.” Staff similarly noted that “when we switched to more of a hybrid telephone visit, people really liked how accessible and easy it was.”

Digital supports also became important implementation adaptations. Online laboratory requisitions and SMS reminders were perceived as helpful for supporting monitoring, appointment attendance, and follow-up. Although some clients experienced minor challenges printing requisitions or accessing online systems, these issues were generally described as manageable rather than prohibitive.

Another important adaptation involved changing the clinic's language and branding. The clinic moved away from language that explicitly targeted “gay men” and adopted the broader and more inclusive name “PrEP clinic.” This change helped communicate the clinic's relevance to a wider range of populations who may benefit from PrEP, including people who may not identify with earlier, more narrowly framed messaging.

#### Acceptability and Appropriateness of Services

Clients consistently described the clinic as safe, nonjudgmental, confidential, and supportive. The location of PrEP services within a sexual health clinic appeared to enhance acceptability by creating a setting where clients felt staff were knowledgeable, prepared, and comfortable discussing sexual health. The specialized nature of the clinic also contributed to perceived appropriateness. Clients contrasted the PrEP clinic with other health care settings, such as walk-in clinics, where they had experienced or anticipated stigma. One client mentioned: “You could talk to anybody like without the taboo of like, oh, they're going to judge me. They're going to give me the look or stuff like that. Yeah, yeah, that was really important.”

For some clients, this affirming environment was particularly meaningful given the history of stigma affecting queer communities. As one client stated, “The queer community has always been immersed in that shame … stigma, taboo and all of that. So having … a clinic that is for us … makes a whole difference.”

#### Determinants

Several CFIR-related determinants shaped implementation of the PrEP clinic model. At the level of intervention characteristics, staff emphasized the adaptability of PrEP delivery within the sexual health clinic setting. Established clinical guidelines, standardized medical directives, and clear prescribing pathways supported fidelity to core components while allowing flexibility in how services were delivered. One staff member noted, “This program has really good written standard operating procedures on how everything operates.” This adaptability became especially important during the COVID-19 pandemic, when the clinic incorporated telephone-based care and temporarily restructured staff roles to maintain service continuity.

Implementation fidelity was supported by inner setting factors, including formal training, shadowing, written policies and protocols, and support from experienced colleagues. Public health residents and structured nursing rotations further contributed to knowledge transfer and service continuity. One staff described how direct training helped them understand the operational details of the clinic:

“I had [staff] as my trainer and I was first trained in [other clinics], so I knew all about appointments, how to make them, how to print off, And then when she felt I was comfortable, that's where she trained me on the every two weeks … You gotta look ahead, book and call those people, get their appointments through their text messages, make sure they know if it's in person or if it's on the phone.” (Staff)

The OSCAR electronic medical record system (CFIR: physical resources/structure) further supported workflow efficiency and access to client information. As one staff member explained, “OSCAR is organized and user-friendly … good functioning around information access.” A dedicated PrEP nurse lead (CFIR/human resources) was also identified as central to coordinating daily operations, supporting protocol adherence, and enabling flexibility without compromising continuity. One staff member shared: *“*That's been a solid piece too, to have a nurse that is the lead and then other nurses that are taking on some of that […] responsibility at a time.”

At the level of individual characteristics, provider self-efficacy and clinical confidence were important for implementation. Staff described feeling prepared to deliver PrEP counseling, initiate conversations about sexual health, troubleshoot side effects, and adapt communication to diverse client needs. Confidence was strengthened through prior public health experience, trauma-informed training, informal mentorship, and hands-on learning. One staff member explained, “I just kind of did it naturally. I think it helps because of my education background … I’m very much used to working in this trauma-informed framework.”

Despite these facilitators, structural barriers at organizational level (inner setting) affected implementation. Because pharmacy services were not available onsite, prescription-related issues, such as prescriptions not being received by fax or delays confirming medication dispensing, required repeated follow-up from staff. Laboratory processes also created challenges because bloodwork needed to be completed off-site. Clients were responsible for managing requisitions, printing forms when needed, booking appointments, and completing tests before follow-up visits, which sometimes delayed monitoring and increased administrative burden for staff (inefficiencies).

Staff also reflected on expanding on-site laboratory services to improve monitoring and reduce administrative burden. While participants felt this could improve adherence and reduce follow-up, they also noted the additional staffing, supplies, infrastructure, and funding required. One staff member explained, “We’d probably have better compliance … maybe if we did the blood work here, and it would be less admin time … But then you think of the time and supplies in clinic to provide that service.”

Finally, the clinic's supportive and nonjudgmental culture contributed to the perceived acceptability and appropriateness of the model. Staff and clients described the clinic as respectful of client autonomy and supportive of informed decision-making. This culture appeared to facilitate client engagement and normalize conversations about PrEP, sexual health, and prevention (see effectiveness).

### Reach

Staff clearly distinguished between populations the clinic was successfully reaching and those who remained underrepresented. Across interviews, participants reported that the clinic primarily engaged more socially advantaged clients, particularly gbMSM with insurance coverage, stable housing, and the ability to self-advocate and navigate health care systems. These perceptions were consistent with clinic record data, which showed that most clients were cisgender men, with limited representation of other populations who may benefit from PrEP. As one staff member explained, “The other populations that we are reaching are the ones that can self advocate and follow through and critically think and don’t need as much support.” Another staff member reflected on whether the clinic was reaching those with the greatest prevention needs: “I’ve often wondered … where are the people who are at highest risk accessing PrEP? … I think a lot of our clientele has fairly low scores.”

However, the clinic's specialized sexual health focus facilitated reach among some clients who had experienced or anticipated discrimination elsewhere.

“I think one of the main things with the, with the public health was the lack of judgement because like even walk in clinics and stuff, you know, you feel the eyes, you feel the, the, the kind of the stigma around it. Whereas it's a clinic specifically for that.” (Client)

These reflections point to an important equity issue: while the clinic was accessible and acceptable to many clients, those most affected by social and structural vulnerability may have remained less likely to enter or remain engaged in the PrEP pathway.

### Determinants

At the level of intervention characteristics, staff identified limitations in the clinic's use of standardized eligibility and risk-assessment tools. Although the HIRI-MSM tool was viewed as evidence-based, staff questioned whether it adequately captured the prevention needs of populations such as people who use drugs and transgender women. One staff member explained that although the tool had been “deemed a very effective tool from a research standpoint,” it may not fully include these populations, meaning the clinic “may be missing out on other pieces to that risk evaluation.” From an equity perspective, rigid reliance on standardized risk scores may unintentionally narrow eligibility and under-recognize risk among groups whose HIV vulnerability is shaped by social and structural conditions not fully captured by the tool. Staff recommended refining risk assessment tools to better capture the lived realities of people who inject drugs, transgender women, and serodiscordant couples.

Financing structures were among the most consequential outer-setting barriers to equitable reach. Staff repeatedly emphasized that, while clients did not need insurance to consult with clinic nurses or physicians, actual PrEP access was often contingent on private insurance, provincial coverage, or the ability to pay. As one staff member stated, “We actually have no other funding source … it's only based on people having benefits or being able to afford it.” Clients also raised cost as a concern during initial counseling, alongside questions about side effects and kidney monitoring. These funding barriers disproportionately affected uninsured and underinsured individuals, including sex workers, people who use drugs, transgender women, and newly arrived residents. As one staff member explained:

“So for individuals that land in our labs, most of them have benefits and for those who don't, we basically leave it up to them to sort of source out how they're going to afford it or how they're going to find the, the, the programs that exist to help them. So, umm, that's where it gets tricky.”

Thus, although the clinic reduced consultation and provider-access barriers, medication affordability remained an externally imposed limitation on reach. However, the clinic still filled an important gap by allowing uninsured or newly arrived residents to speak with nurses and physicians about PrEP without requiring OHIP or private insurance. One staff member noted, “They don’t need any type of insurance to see a physician or our nurses for at least a chat about HIV PrEP … we don’t.” This function positioned the clinic as a partial safety net, even when medication access remained constrained.

Geography (CFIR: outer setting) also shaped reach. Clients and staff raised concerns that the clinic may under-reach people living in rural or remote areas, people without reliable transportation, and individuals outside the immediate catchment area. One client suggested that medication delivery or mailed prescriptions could improve access for people who “can’t leave the home,” “can’t get out to remote locations,” or “live in remote locations.” At the same time, the physical location, hours of operation, and public visibility of the clinic could limit access for others. Thus, structural and logistical features (CFIR: inner settings) may still discourage or prevent access among people with privacy concerns, work constraints, transportation barriers, or discomfort entering a visibly identifiable public health setting. One staff member noted that:

“There's some stigma around coming to our building, it's very, um, modern and not maybe in a place that is comfortable for certain people to, to enter our building. So that does impact who could access our services even the time of day that we offer services because we have no evening clinics, right.”

In this regards, staff and clients suggested adaptations included extended clinic hours, weekend appointments, mobile outreach, and venue-based services to accommodate individuals with unstable housing, irregular work schedules, or limited mobility.

“If I could change, I would want to have a whole day appointment to factor people who who, who can be seen in the morning and some people can be seen in the afternoon. I know some patients have asked why we don't have for the weekend would be good to have a weekend, but I just don't know if the agency would be willing to, you know, to, to pay that extra for, for, for that service.” (Staff)

Participants highlighted the importance of strengthening partnerships with community-based organizations, student wellness centers, queer-serving agencies, harm reduction programs, and sex worker advocacy groups to equity reaching those underserved by the clinic. Engagement strategies suggested by staff included physically bringing services into community spaces: “So if you were to reach that client, you may need to take the clinic to them, like physically take the services to them.” Staff also proposed establishing communities of practice with other PrEP providers to improve knowledge exchange and inclusive engagement strategies.

“I think the other piece that could happen potentially is to […] look at even like a community of practice type approach with others that are doing Prep […] and touch base more about specifically talking about PrEP and how to reach clients in a more inclusive way.” (Staff)

Primary care capacity was another important determinant of reach. Staff and clients noted that many external primary care providers were unfamiliar with PrEP or unwilling to prescribe it, while many clients lacked a regular family physician altogether. In this context, the PrEP clinic functioned as an alternative access point for clients who could not rely on primary care for HIV prevention.

“A lot of healthcare providers may or may not be super knowledgeable … and a lot of people don’t have primary care providers. A lot of our clientele is transient or have moved here recently, so they obviously wouldn’t have a family doctor to go to.” (Staff).

However, limited PrEP knowledge among external providers also constrained referral pathways and placed greater responsibility on the sexual health clinic. Normalizing PrEP conversations across routine STI screening, quick testing visits, and primary care consultations was also described by staff as a critical engagement strategy: “We're trying to introduce that into our conversations in testing and screening with even just with our community providers just to demystify it and make it part of the conversation.” Training primary care providers and proactively integrating PrEP discussions into standard care pathways were seen as mechanisms to strengthen adoption beyond the sexual health clinic and improve reach at the system level, as mentioned by one staff: *“*Are you thinking about putting them on PrEP? … I’ve emailed a little package out to other doctors.”

At the individual level, client knowledge, confidence, and ability to navigate health systems influenced reach and engagement. Some clients demonstrated high levels of health literacy, familiarity with STI testing, and ability to search for information about PrEP, dosing options, monitoring requirements, and related prevention tools such as DoxyPEP. One client described “Googling DOXYPEP” and learning about medication to help prevent STIs after condomless sex. Staff mentioned that clients with greater health system navigation skills may have been better positioned to find, understand, and use the clinic's services. Conversely, clients with lower health literacy, fewer digital resources, or less confidence advocating for themselves may have been less likely to access the clinic.

“The population served is majority men having sex with men … knowledgeable … working class… they have insurance.” (Staff)

“The other populations that we are reaching are the ones that can self-advocate and follow through.” (Staff)

“Our clientele … are very familiar with chlamydia, gonorrhea, syphilis … the importance of testing.” (Staff)

Finally, social networks and community knowledge acted as facilitators of reach. Informal peer support helped normalize PrEP use, reduce uncertainty, and direct clients toward the clinic. One client described how “a friend back home who was also on PrEP talked me through it,” after which they conducted their own research. These peer pathways suggest that community networks can play an important role in expanding reach, particularly when formal referral systems are limited or when clients are hesitant to seek PrEP through general health care settings.

### Effectiveness

#### Clinical Outcomes

Effectiveness was reflected not only in biomedical HIV prevention, as reported in separate analyses of clinic records, but also in improvements in client well-being, confidence, and engagement in care. Clients consistently described PrEP as reducing anxiety about HIV acquisition and increasing comfort in sexual relationships. For many, PrEP provided psychological reassurance that extended beyond clinical protection. As one client explained,

“I cannot underestimate the positive mental health impact … just knowing that as long as I continue to take my medication, this is not something I have to be concerned about is huge for someone who approached sexuality with so much fear.”

Clients also framed PrEP as helping them meet their personal prevention goals. One client stated, “For me the goal was just to be as safe as possible. And I think I did achieve that.” From the staff perspective, effectiveness was similarly reflected in clients’ increased confidence in sexual health decision-making and greater comfort discussing sexuality openly.

Both staff and clients described PrEP as highly acceptable as a medication, with few adverse effects reported (safety outcome). This perception was consistent with clinic record data showing positive clinical outcomes and continued engagement with PrEP care. Staff described many clients as “very active in their healthcare and getting their blood work done and showing up to their appointment.”

However, staff also identified a potential unintended consequence of PrEP effectiveness: increased risk of other sexually transmitted infections associated with reduced condom use. One staff member explained, “Definitely very effective at preventing HIV. But I think an unanticipated byproduct of the success of PrEP comes at the expense of a higher rate of other infections because people are using barriers less.”

#### Flexibility and Responsiveness

Effectiveness was further shaped by the clinic's flexibility and responsiveness to client needs. Staff described efforts to accommodate client schedules and troubleshoot coordination challenges related to off-site laboratories, pharmacies, prescriptions, and follow-up testing. The hybrid model was particularly important in reducing the burden of repeated in-person visits. Staff noted that, after the transition to telephone-based follow-up, many clients appreciated the efficiency of quick visits over the phone for the vast majority of visits: So when we switch to more of a kind of hybrid telephone visit, a lot people really like the how it's like, yeah, they're quick visits over the phone for the vast majority of visits. Digital supports, including online laboratory requisitions and SMS reminders, facilitated monitoring, follow-up, and appointment attendance. However, staff recognized that these adaptations still required a certain level of digital access and literacy.

Although the hybrid model improved convenience, some clients still experienced limitations in appointment flexibility. Restricted Monday afternoon clinic hours were described as challenging for people with rigid work schedules. One client suggested that the clinic could be more accommodating “if there was a way to make it earlier or extend it a little bit … with work schedules.”

#### Operational Efficiency and Workflow Challenges

Participants linked effectiveness to operational efficiency. Sustaining engagement required ongoing coordination of laboratory requisitions, prescriptions, pharmacies, and follow-up appointments. While logistical issues were generally minimal, both clients and staff described delays related to prescriptions, bloodwork, and pharmacy communication as disruptive and time-consuming. For example, one client reported, “There were issues at the pharmacy … I had to call back twice and get them to resend it … very minimal, but those things happen.”

Staff identified opportunities to optimize workflow and improve efficiency through greater role differentiation and task sharing. They suggested that some follow-up activities currently handled by physicians could be delegated to nurses within their scope of practice. One staff member noted, “We’ve talked about doing nurse-led PrEP … some of the stuff the physicians are doing, we as nurses could do within our scope … maybe divvy out the roles differently.”

#### Determinants of Effectiveness

Several CFIR-related determinants shaped the effectiveness of the PrEP clinic model. At the level of intervention characteristics, the hybrid model improved fit with clients’ lives and clinic workflow by reducing travel, time commitments, and repeated in-person visits. Staff also described telephone follow-up as more efficient for both physicians and clients, contributing to retention of the model after the pandemic.

Flexibility in PrEP modalities also contributed to effectiveness and engagement. Staff described on-demand or intermittent PrEP as especially useful for clients who had difficulty adhering to daily medication or who faced cost barriers. As one staff member explained, “For some of the people who can’t afford it or have a difficult time being adherent to daily—being able to offer them … on-demand PrEP has been great.” This flexibility allowed counseling and prescribing to better align with clients’ circumstances, sexual practices, adherence capacity, and financial constraints.

At the inner setting and cultural climate levels, the clinic's nonjudgmental and specialized sexual health environment strengthened client trust and engagement. They valued staff who were knowledgeable about PrEP and who made it comfortable to ask difficult or sensitive questions. One client said, “I definitely prefer the sexual health clinic just to find a safer space and more knowledgeable care.”

Outer setting and structural determinants limited effectiveness for some clients. As described under reach, geography, transportation, and medication affordability continued to shape clients’ ability to initiate and sustain PrEP care. One client explained, “The cost is a big issue. Before I found a medication alternative, I was ready to take the plunge of $750 every three months.” In the absence of universal PrEP medication coverage, staff spent considerable time helping clients identify possible funding options, troubleshoot insurance issues, and navigate access pathways. While this support helped some clients remain engaged in care, it also created inefficiencies and reduced time available for direct clinical work. Staff described this also as a sustainability challenge because the clinic's capacity to expand services was limited by the ongoing labor required to address these gaps.

### Maintenance

The PrEP clinic has delivered services since 2018, suggesting sustained organizational commitment to PrEP implementation. Qualitative narratives from both staff and clients provide insight into how maintenance has been achieved over time. Clients described sustained engagement with PrEP and strong adherence across extended periods. One client explained, “The goal was to have healthy sexual encounters … I really stuck to the PrEP program. I think I missed like one dose, maybe two doses over the 3 years that I was taking it.” Staff similarly described PrEP delivery as increasingly embedded in routine sexual health workflows. Over time, the clinic moved from an early period of adaptation and “growing pains” toward a more stable and standardized process. One staff member noted, “There's been various changes throughout the years … but for the last year or so, I think the process has stabilized and everyone kind of understands what the process is.”

#### Determinants of Maintenance

Several CFIR-related determinants shaped the maintenance and long-term sustainability of the PrEP clinic. At the level of the outer setting, staff described evolving external conditions that may require ongoing adaptation. This included uncertainty related to public health unit mergers, shifting organizational priorities toward emerging health issues, the introduction of new PrEP modalities such as long-acting injectable formulations, and persistent gaps in insurance coverage for PrEP medication and laboratory monitoring.

Workforce shortages in the broader primary care environment also constrained sustainability. Staff noted that limited availability of community-based providers who were knowledgeable about, willing to prescribe, or able to manage PrEP reinforced the clinic's central role in ongoing service delivery. As a result, the clinic carried a disproportionate share of PrEP care in the region. This external provider gap intensified internal capacity pressures and reduced opportunities to decentralize PrEP management across primary care or community settings.

Within the inner setting, staffing and resource constraints emerged as major determinants of maintenance. Clinic operations relied heavily on a single physician with historical knowledge of the program and responsibility for prescribing, which staff identified as a key sustainability vulnerability. One staff member explained:

“The reality is there is only one physician … and if it's a physician-led clinic, which it truly is, then you need more physicians, then you get into contracting and more cost. And I don’t think what we’re earning off OHIP billing is probably covering the full cost.”

The reliance on a single prescribing physician prompted discussions about alternative models, including nurse-led PrEP clinics. Navigation support—such as case managers assisting with insurance applications, subsidy programs, and follow-up care—was identified as a key quality improvement strategy to enhance reach and sustain engagement: “We could do a better job recruiting the people who are at greatest risk by equipping ourselves with knowledge to find funding” (Staff).

The possibility of a nurse-led PrEP model was therefore discussed as a strategy to strengthen sustainability and reduce dependence on physician capacity. However, staff noted that some clinical responsibilities may fall outside existing nursing scope or current medical directives, particularly aspects of laboratory interpretation and renal monitoring. One staff member explained, “I know we’ve pushed for a nurse led PrEP, but … there are things that I don’t think a medical directive will cover us for … creatinine levels … are required for PrEP.” This highlights the need for clear role definitions, expanded medical directives where appropriate, and sufficient clinical oversight to ensure that task-sharing models maintain safety, quality, and continuity.

Additional operational challenges affected maintenance as the clinic evolved. Staff described delays in formalizing policies after pandemic-related changes and the loss of institutional knowledge following the departure of a clinical nurse facilitator. These changes created gaps during a period when the model was adapting from emergency pandemic processes to a more stable hybrid approach. Such challenges illustrate how sustainment depends not only on whether a program continues, but also on whether the infrastructure, documentation, staffing, and institutional knowledge needed to support consistent delivery are maintained.

Funding constraints were repeatedly identified as limiting the clinic's ability to expand staffing, outreach, and service capacity. Staff described an ongoing tension between increasing reach and preserving quality within existing resource limits. As one staff member summarized, “We could increase reach … but what is the capacity for that?”. This tension is central to maintenance: expanding the clinic may improve population-level impact, but without additional resources, expansion could overburden staff, weaken fidelity, or reduce responsiveness.

Participants mentioned that sustainable expansion of PrEP services requires intentional planning aligned with clinic capacity and long-term strategic goals. A central recommendation was securing dedicated funding mechanisms**—**either through public financing reforms or insurance adjustments—to cover PrEP medications and laboratory costs for uninsured individuals, as mentioned by one staff “Other than the fact that we have no funding for PrEP medications … if you could have your clinic tied to a funding source.” Participants stressed the need for clear program goals: whether the clinic intends to focus on a defined high-risk population or expand broadly. Aligning capacity, outreach, and staffing models with these goals was viewed as essential for sustainable adoption and maintenance: “Are we hitting certain types of target needs? … It helped us set goals for the future” (Staff).

Despite these challenges, sustained leadership support and community connection were important facilitators of maintenance. Leadership and external engagement helped protect the clinic through major disruptions, including the COVID-19 pandemic and uncertainty related to regional public health restructuring. Staff also emphasized that maintaining the clinic strengthened the organization's relationship with gbMSM communities. One staff member explained, “I think the benefit of maintaining our clinic is, again for that connection with the community and for that reputational link with the [gbMSM] communities.” This connection reinforced the perceived value of sustaining the clinic, not only as a PrEP access point but also as a trusted public health service for communities historically affected by HIV-related stigma and inequities.

Ongoing evaluation and structured reflection (CFIR: process) were described as essential to sustaining the clinic over time. Staff emphasized the need for improved data entry systems, standardized documentation, and performance monitoring to strengthen institutional memory and reduce reliance on informal knowledge: “Is our client health stable? … What's the general demographics?”*.* Continuous evaluation was seen as critical to identifying implementation gaps, monitoring changes in Reach, and recalibrating outreach strategies.

“I mean, I would love to see eventually that we can have that more, um, systematic so that we have a way to extract that information with, you know, whether it's Power BI or, um, just so that we can have it more routinely done on a quarterly basis so that we don't have to constantly be manually doing all of that work.” (Staff)

## Discussion

This evaluation examined the adoption, implementation, reach, effectiveness, and maintenance of a PrEP clinic embedded within sexual health services in a mid-sized Ontario public health unit. Previous analyses of the clinic demonstrated high levels of client engagement, strong clinical effectiveness, including zero HIV seroconversions among clients in care, and positive client perceptions of service quality, fidelity, and acceptability.^23^ In this paper, we extend those findings by examining the implementation determinants that shaped the delivery of PrEP in this setting, with particular attention to fidelity, effectiveness, sustainability, and equity across the PrEP care cascade. By integrating RE-AIM with CFIR and a health equity lens, this evaluation provides a more comprehensive understanding of how the clinic model was adopted, adapted, and sustained over time, and how structural and organizational factors influenced its capacity to reach diverse populations who may benefit from PrEP.

### RE-AIM and CFIR

The use of CFIR and RE-AIM as frameworks provide insight into both what was achieved and why certain aspects of implementation were successful or constrained. Stating with adoption, key determinants were: characteristics of intervention, recognition of unmet HIV prevention needs, strong belief in PrEP as an effective and necessary intervention, alignment with public health mandates, and the perception that implementation would be feasible, simple and not too costly; outer setting factors including unmet community need, limited regional access to PrEP providers, local gaps in PrEP access, and limited number of providers in the region; and inner setting dynamics, including supportive leadership, a collaborative organizational culture, informal mentorship, and a positive learning climate; determinants consistent with findings in other settings.^19,26,32,33^

Implementation and sustainment have been facilitated by key inner setting domains, the organization's strong adaptive capacity, particularly through the rapid introduction of telehealth (mostly phone call) in response to COVID-19 restrictions and the appointment of a dedicated PrEP nurse lead. This adaptability capacity has been identified as key factor differentiating those able to adopt PrEP, by prioritizing services.^19,22^ In our case study, the shift to telehealth enabled continuity of care during system disruptions while improving convenience and accessibility for many clients. Importantly, these findings provide evidence that a hybrid telehealth PrEP model, requiring only one in-person visit per year, besides of being feasible, acceptable and with high fidelity, may be effective. This is particularly notable within the provincial context, where online PrEP provision exists (eg, GoFreddie, PrEP start) but has not been formally evaluated. Similarly, designating a PrEP champion or coordinator consolidated expertise, strengthened workflow consistency, and enhanced follow-up processes. The presence of a dedicated PrEP lead has been identified in other settings as key facilitators of client engagement, implementation fidelity, and sustained effectiveness.^34,35^ The experience, knowledge, and strong commitment of clinic staff also contributed substantially to consistent implementation and sustained engagement.^22^

With respect to reach, the clinic predominantly serves gbMSM, a population that remains at elevated risk of HIV infection in Ontario and for whom PrEP is a key prevention strategy.^36^ The clinic provides a relatively low-barrier model for many gbMSM by offering nonjudgmental, supportive, and specialized sexual health care. However, staff and clients described reach as uneven. Despite this, women, transgender individuals, sex workers, people who use drugs, unhoused individuals, students, and people living in rural or geographically distant areas appeared to be underrepresented. This pattern aligns with Canadian and provincial evidence documenting persistent inequities in PrEP access among populations who may benefit from HIV prevention but face intersecting structural barriers, including cost, stigma, geography, and limited access to affirming providers.^12‐14^ Experiences of other sexual health clinics in Canada demonstrated the same trends observed in this case study.^37^ Thus, while sexual health clinics can serve as important and trusted access points for PrEP, they often lack the structural capacity, resources, and cross-sector integration required to fully address the social and contextual barriers that constrain equitable access across the PrEP cascade.^19^

The results pointed out that the underrepresentation of populations in reach can be more fully understood through CFIR constructs, particularly within the outer setting and inner setting domains. Outer setting factors, including patient needs and resources, help explain how structural barriers (eg, medication cost, insurance coverage, geographic access, and stigma) shape who is able to engage with clinic-based PrEP services. At the same time, inner setting characteristics, including limited clinic hours, constrained outreach capacity, and a predominantly demand-driven service model, further influence access. Barriers appear particularly pronounced in rural contexts, where limited provider availability compounds broader structural challenges such as substance use, the needs of Indigenous communities, housing instability, and co-occurring mental health conditions.^22,38^

Nevertheless, staff described an ongoing “tension for change,” reflecting a strong commitment to expanding reach to equity-deserving populations without corresponding increases in staffing or financial resources. This tension appears closely linked to broader contextual constraints, including the absence of universal provincial coverage for PrEP medications and laboratory monitoring, limited funding for additional personnel, and a shortage of PrEP prescribers outside the organization; consistently identified as barriers to PrEP adoption and determinants of inequities in access.^19,22^

Contrary to experience with reach, early effectiveness outcomes based on both program indicators and client-reported experiences, were strong, with no HIV seroconversions observed during follow-up and high levels of client satisfaction, nor differences in engagement by sociodemographic characteristics collected in clinic records.^23^ More importantly, clients consistently reported reduced anxiety related to HIV acquisition, improved sexual well-being, and greater confidence in prevention decision-making. From a CFIR perspective, these outcomes seem to be related to inner setting factors, including a supportive and nonjudgmental care environment, the fidelity of the intervention, and individual-level characteristics such as client motivation and health literacy, and level of knowledge and continuing capacity of staff in the clinic.

### Actionable Recommendations

Participants offered several practice-informed recommendations that aligned with RE-AIM outcome dimensions, reflected key CFIR implementation determinants, and were consistent with existing evidence. These recommendations may inform PrEP implementation across public health, sexual health, and primary care settings. To facilitate adoption in other settings, implementation strategies should therefore address both the perceived value of PrEP and the organizational infrastructure required to deliver it. Clinics may need standardized protocols, staff training, prescribing pathways, laboratory monitoring processes, medication-access guidance, and effective referral systems. In lower-resource, rural, or nonspecialized settings, adoption may require additional adaptations such as shared-care arrangements, virtual specialist support, nurse practitioner-led prescribing, or partnerships with pharmacies and community organizations.^39,40^ These strategies can help reduce dependence on a single clinic or provider and support broader system-level adoption.

The evaluation suggests that several components should be preserved because they are central to fidelity, acceptability, and effectiveness. These include eligibility assessment using both standardized tools and clinical judgment, adherence and side-effect counseling by a lead nurse, regular STBBI testing, medication-access support, and affirming, nonjudgmental sexual health care. From a CFIR perspective, these components reflect the core functions of the intervention and should be protected during adaptation. Other components can be adapted to local context. The specific balance between telephone, virtual, and in-person visits may vary depending on geography, staffing, laboratory access, client preferences, and organizational capacity. Clinic hours, referral pathways, staffing roles, and follow-up systems may also need to be modified. The key is to preserve the function of the model—safe, timely, affirming, and clinically appropriate PrEP care—while adapting the form to fit local conditions.

The literature suggests that improving equitable access to PrEP requires multilevel strategies that combine clinic-level adaptations with broader structural supports. Evidence supports integrating PrEP discussions into routine STI screening, primary care, and sexual health visits^39,41^; recent Canadian guidelines also recommend offering PrEP to adults and adolescents who request it, reinforcing the need for broader and less restrictive access models.^17^ Public coverage for PrEP medication is particularly important to reduce financial barriers, increase uptake, and decrease the administrative burden associated with insurance navigation.

Several adaptations could further improve reach and retention. Mobile clinics, embedded within existing outreach and testing programs, could extend PrEP access to underserved populations by reducing transportation barriers, increasing flexibility, and supporting ongoing engagement.^42,43^ Clinics could also begin establishing pathways for long-acting injectable PrEP, which may improve sustained protection for individuals facing adherence challenges with daily oral regimens.^44^ In parallel, providing navigation support for insurance and drug coverage applications (until no-cost PrEP is available in Ontario), alongside structured follow-up, can mitigate financial and administrative barriers that undermine continuity of care. Navigation roles could be filled by trained staff or peers, drawing on competencies used in HIV case management models that have demonstrated reach and effectiveness in other jurisdictions.^45‐47^ Finally, sexual health clinics can pursue operational innovations—such as digital eligibility screening, virtual counseling options, and automated reminders—to strengthen fidelity, reduce administrative burden, and improve clinic efficiency.

Expanding telehealth, mobile services, and outreach strategies for PrEP delivery are also strategies that could increase equity in reach. Historically underserved populations—including transgender people, sex workers, people who use drugs, students, people experiencing housing instability, and rural residents—require targeted approaches through partnerships with community-based organizations, harm reduction programs, student health services, and queer-serving agencies.^44^ Virtual and hybrid PrEP models can reduce transportation, scheduling, and stigma-related barriers, particularly in rural areas or settings with provider shortages. However, telehealth alone is unlikely to address inequities unless paired with complementary supports such as free or subsidized medication, insurance navigation, proactive follow-up, and care coordination. Without these complementary components, telehealth alone may not overcome financial and administrative barriers that limit uptake and retention.

Increasing the number and diversity of PrEP providers is essential for broader system-level adoption. Participants recommended strengthening primary care provider training and integrating PrEP education into routine care. Mixed or hybrid models—including shared-care arrangements and virtual consultation supports—have demonstrated potential to increase provider knowledge and prescribing confidence across diverse practice settings.^48^ However, expanding adoption also requires attention to organizational culture, leadership support, and sustainable funding structures.^19^ Sexual health clinics are recovering from restrictions of the COVID-19 pandemic and leadership support, and innovative funding structures will be essential to increase adoption of PrEP and its sustainability over time.

While some sexual health clinics have opted not to provide PrEP on-site due to the perceived availability of external prescribers, this model does not appear to function consistently in practice.^19,22^ Referral pathways are often informal and insufficiently structured, creating uncertainty around linkage to care and continuity of follow-up. Previous work has similarly shown that reliance on external referral systems may weaken engagement and reduce successful PrEP initiation and retention.^49,50^ Providing PrEP-on-site in sexual health clinics may represent an acceptable and feasible delivery approach in similar mid-sized public health settings, although this recommendation coming from our results should be interpreted considering the study's scope and context.

In Ontario, nurse-led and nurse practitioner-supported PrEP models have already been implemented, demonstrating the feasibility of task-shifting within public health and community-based settings.^20^Community pharmacy-based PrEP is one promising option, particularly where primary care access is limited.^51^ Pharmacist-led prescribing and monitoring, combined with rapid HIV/STI testing, could support integrated “test-and-prevent” pathways and expand access beyond traditional clinic-based models.

### Limitations

This study has several limitations. While all experienced staff were included in interviews, the qualitative sample of clinic clients was relatively small and based on purposive, self-selected recruitment, which may limit the diversity of perspectives captured and introduce selection bias. Client interview participants represented a subset of individuals who engaged with the clinic, and thematic saturation may not have been achieved, particularly across diverse or underrepresented subgroups. As a result, participants may reflect individuals who were more engaged or satisfied with the clinic and may under-represent those who were disengaged from care or facing greater structural barriers, including unstable housing or limited access to communication technologies. In addition, although multiple data sources were used, the present analysis is primarily qualitative, with quantitative data providing contextual support. Findings are also based on a single clinic within one regional context, which may limit generalizability to other settings. However, the use of implementation and evaluation frameworks, including RE-AIM, CFIR, and HEIF, strengthened analytic rigor and provides a useful approach for evaluating other PrEP programs, identifying equity-related implementation gaps, and generating recommendations for adapting PrEP delivery across diverse public health and sexual health clinic settings.

### Conclusion

While RE-AIM guided the assessment of implementation outcomes, applying CFIR enabled deeper understanding of the contextual factors shaping these outcomes. Together, these frameworks provide insight into both what was achieved and why certain aspects of implementation were successful or constrained. This study demonstrates that sexual health clinics can successfully adopt, implement, and sustain high-quality PrEP services within existing public health infrastructures. However, meaningful reductions in HIV-related inequities will require deliberate, equity-centered implementation strategies that extend beyond clinic walls. Internal excellence in service delivery—while necessary—is insufficient on its own. Sustainable and equitable impact depends on structural reforms, strengthened community partnerships, and proactive outreach strategies to ensure that populations at highest risk are not the least reached. In particular, provincial-level reforms to ensure stable and universal funding for PrEP medications, laboratory monitoring, and service delivery are critical structural levers to expand adoption, reduce administrative barriers, and mitigate inequities in access. Without such systemic changes, even well-functioning clinics may struggle to translate local implementation success into broader population-level equity gains.

## Supplemental Material

sj-docx-1-jia-10.1177_23259582261458932 - Supplemental material for Determinants of Adoption, Implementation, Reach, and Sustainability of PrEP Services in a Sexual Health Clinic in Canada: A Qualitative Analysis Using CFIR and REAIM Supplemental material, sj-docx-1-jia-10.1177_23259582261458932 for Determinants of Adoption, Implementation, Reach, and Sustainability of PrEP Services in a Sexual Health Clinic in Canada: A Qualitative Analysis Using CFIR and REAIM by Emma Nagy, Beatriz Alvarado, Miriam Kamotho, Nicole Szumlanski, Tianxiu Hugh Guan and Jorge Martinez-Cajas in Journal of the International Association of Providers of AIDS Care (JIAPAC)

sj-docx-2-jia-10.1177_23259582261458932 - Supplemental material for Determinants of Adoption, Implementation, Reach, and Sustainability of PrEP Services in a Sexual Health Clinic in Canada: A Qualitative Analysis Using CFIR and REAIM Supplemental material, sj-docx-2-jia-10.1177_23259582261458932 for Determinants of Adoption, Implementation, Reach, and Sustainability of PrEP Services in a Sexual Health Clinic in Canada: A Qualitative Analysis Using CFIR and REAIM by Emma Nagy, Beatriz Alvarado, Miriam Kamotho, Nicole Szumlanski, Tianxiu Hugh Guan and Jorge Martinez-Cajas in Journal of the International Association of Providers of AIDS Care (JIAPAC)

## References

[1] O Murchu E Marshall L Teljeur C , et al. Oral pre-exposure prophylaxis (PrEP) to prevent HIV: A systematic review and meta-analysis of clinical effectiveness, safety, adherence and risk compensation in all populations. BMJ Open. 2022;12(5):e048478. doi:10.1136/bmjopen-2020-048478 PMC9096492 35545381

[2] Canada PHA of. HIV in Canada: 2023 surveillance highlights. November 29, 2024. Accessed February 18, 2025. https://www.canada.ca/en/public-health/services/publications/diseases-conditions/hiv-2023-surveillance-highlights-infographic.html

[3] Canada PHA of. HIV-PrEP use and HIV-PrEP-to-need ratio in nine Canadian provinces, 2018–2021, CCDR 51(1). January 2, 2025. Accessed February 25, 2025. https://www.canada.ca/en/public-health/services/reports-publications/canada-communicable-disease-report-ccdr/monthly-issue/2025-51/issue-1-january-2025/hiv-prep-use-need-ratio-nine-canadian-provinces-2018-2021.html

[4] PHAC. *Trends in HIV Pre-exposure Prophylaxis Use. An Analysis of Prescription Data in Eight Canadian Provinces, 2016–2020. 2021.* Public Health Agency of Canada.; 2021. Accessed on 15 October, 2021. https://www.canada.ca/en/public-health/services/publications/diseases-conditions/hiv-trends-pre-exposure-prophylaxis-canadian-provinces-2016-2020.html

[5] Popovic N Yang Q Archibald C . Trends in HIV pre-exposure prophylaxis use in eight Canadian provinces, 2014–2018. Can Commun Dis Rep. 2021;47(5-6):251‐258. doi:10.14745/ccdr.v47i56a02 34220349 PMC8219062

[6] Ontario HIV Treatment Network. HIV Pre-Exposure Prophylaxis (PrEP) in Ontario, 2022. OHTN; 2022.

[7] OHTN. *HIV Pre-Exposure Prophylaxis (PrEP) in Ontario,* 2021. Ontario HIV Treatment Network; 2021. https://www.ohtn.on.ca/wp-content/uploads/2023/04/OHTN-PrEP-report-2021-2023APR06.pdf

[8] Alvarado B Kuforiji O Cofie N , et al. Strategies to overcome barriers and enhance PrEP adoption among primary care providers in urban–rural communities outside Canada’s major metropolitan areas. J HIVAIDS Soc Serv. 2026;24(2):154–178. doi:https://doi.org/10.1080/15381501.2025.2572602

[9] Sinno J Daroya E Wells A , et al. “To do so in a patient-centred way is not particularly lucrative”: The effects of neoliberal health care on PrEP implementation and delivery. Soc Sci Med. 2024;347:116749. doi:10.1016/j.socscimed.2024.116749 38492264

[10] Gaspar M Tan DHS Lachowsky N , et al. HIV pre-exposure prophylaxis (PrEP) should be free across Canada to those meeting evidence-based guidelines. Can J Hum Sex. 2022;31(3):309‐313. doi:10.3138/cjhs.2022-0004

[11] Tan DHS Dashwood TM Wilton J Kroch A Gomes T Martins D . Trends in HIV pre-exposure prophylaxis uptake in Ontario, Canada, and impact of policy changes: A population-based analysis of projected pharmacy data (2015–2018). Can J Public Health. 2021;112(1):89‐96. doi:10.17269/s41997-020-00332-3 32529552 PMC7851246

[12] Adam BD Monteza-Quiroz D Hart TA , et al. Inequitable access to PrEP among gay, bisexual, and other men who have sex with men in Canada: A network analysis of social indicators. SSM - Popul Health. 2025;30:101771. doi:10.1016/j.ssmph.2025.101771 40177026 PMC11964654

[13] Ajiboye W Tharao W Owino M , et al. Racial disparities in HIV pre-exposure prophylaxis (PrEP) awareness and uptake among white, black, and indigenous men in Canada: Analysis of data from the I’m ready national HIV self-testing study. Can J Public Health Rev Can Sante Publique. 2025;116(2):243‐253. doi:10.17269/s41997-025-01009-5 PMC12075050 40106208

[14] Orser L Elmekki M Francoeur M O’Byrne P . HIV Prevention for women: Exploring the uptake of pre- and postexposure prophylaxis (PrEP and PEP) among cis and trans women accessing nurse-led HIV prevention services in Ottawa, Canada (PrEP-RN). Can J Hum Sex. 2023;32(3):289‐297. doi:10.3138/cjhs.2022-0039

[15] Palma PA Skakoon-Sparling S Dawson J , et al. Disparities in healthcare, STI testing, and PrEP access among newcomer sexual minority men in Canada’s three largest urban centers. Anal Soc Issues Public Policy. 2025;25(1):e12448. doi:10.1111/asap.12448

[16] Van Uum R Masshadi PE Canani F , et al. Characterizing cis and trans women’s HIV risk and access to HIV prophylaxis in Ontario, Canada. Open AIDS J. 2025;19(1):e18746136387776. doi:10.2174/0118746136387776250825202101

[17] Tan DHS Hull MW Onyegbule SO , et al. Canadian Guideline on HIV pre- and postexposure prophylaxis: 2025 update. CMAJ. 2025;197(41):E1374‐E1391. doi:10.1503/cmaj.250511 PMC12680393 41326046

[18] Charest M Sharma M Chris A , et al. Decentralizing PrEP delivery: Implementation and dissemination strategies to increase PrEP uptake among MSM in Toronto, Canada. PLOS ONE. 2021;16(3):e0248626. doi:10.1371/journal.pone.0248626 PMC7971529 33735209

[19] Nagy E Alvarado B Rapino C , et al. Factors influencing PrEP adoption in sexual health clinics within Ontario’s public health system: A qualitative study using the consolidated framework for implementation research (CFIR). Front Public Health. 2026;14:1760989. doi:10.3389/fpubh.2026.1760989 42100530 PMC13144111

[20] O’Byrne P Vandyk A Orser L Haines M . Nurse-led PrEP-RN clinic: A prospective cohort study exploring task-shifting HIV prevention to public health nurses. BMJ Open. 2021;11(1):e040817. doi:10.1136/bmjopen-2020-040817 PMC7797243 33414144

[21] Kroch A O’Byrne P Orser L , et al. Increased PrEP uptake and PrEP-RN coincide with decreased HIV diagnoses in men who have sex with men in Ottawa, Canada. Can Commun Dis Rep Releve Mal Transm Au Can. 2023;49(6):274‐281. doi:10.14745/ccdr.v49i06a04 PMC10911788 38440773

[22] Alvarado B Kuforiji O Cofie N , et al. Strategies to Overcome Barriers and Enhance PrEP Adoption Among Primary Care Providers in Urban–Rural Communities Outside Canada’s Major Metropolitan Areas. *Press*. Published online 2025.

[23] Rapino C Nagy E Saeed S , et al. Using the RE-AIM Framework to Evaluate the Reach of Pre-Exposure Prophylaxis Services in a Sexual Health Clinic in Ontario, Canada. *Press*.

[24] Glasgow RE Vogt TM Boles SM . Evaluating the public health impact of health promotion interventions: The RE-AIM framework. Am J Public Health. 1999;89(9):1322‐1327. doi:10.2105/ajph.89.9.1322 10474547 PMC1508772

[25] Damschroder LJ Reardon CM Opra Widerquist MA Lowery J . Conceptualizing outcomes for use with the consolidated framework for implementation research (CFIR): The CFIR outcomes addendum. Implement Sci. 2022;17(1):1. doi:10.1186/s13012-021-01181-5 35065675 PMC8783408

[26] Li DH Benbow N Keiser B , et al. Determinants of implementation for HIV Pre-exposure prophylaxis based on an updated consolidated framework for implementation research: A systematic review. J Acquir Immune Defic Syndr. 2022;90(S1):S235‐s246. doi:10.1097/qai.0000000000002984 PMC10161203 35703776

[27] Piper KN Brown LL Tamler I Kalokhe AS Sales JM . Application of the consolidated framework for implementation research to facilitate delivery of trauma-informed HIV care. Ethn Dis. 2021;31(1):109‐118. doi:10.18865/ed.31.1.109 33519161 PMC7843045

[28] Sales JM Anderson KM Kokubun CW . Application of the consolidated framework for implementation research to facilitate violence screening in HIV care settings: A review of the literature. Curr HIVAIDS Rep. 2021;18(4):309‐327. doi:10.1007/s11904-021-00555-0 33866483

[29] King DK Shoup JA Raebel MA , et al. Planning for Implementation Success Using RE-AIM and CFIR Frameworks: A Qualitative Study. Front Public Health. 2020;8. doi:10.3389/fpubh.2020.00059 PMC7063029 32195217

[30] Woodward EN Singh RS Ndebele-Ngwenya P Melgar Castillo A Dickson KS Kirchner JE . A more practical guide to incorporating health equity domains in implementation determinant frameworks. Implement Sci Commun. 2021;2(1):61. doi:10.1186/s43058-021-00146-5 34090524 PMC8178842

[31] Hsieh HF Shannon SE . Three approaches to qualitative content analysis. Qual Health Res. 2005;15(9):1277‐1288. doi:10.1177/1049732305276687 16204405

[32] Bolívar-Rocha MC Arrivillaga M Camargo-Plazas P , et al. Organizational factors related to the implementation of HIV-PrEP: A qualitative exploration with healthcare managers and providers working in HIV clinics in Colombia. Sex Gend Divers Soc Serv. 2025;37(2):402–427. doi:https://doi.org/10.1080/10538720.2023.2291088

[33] Piper KN Haardörfer R Escoffery C Sheth AN Sales J . Exploring the heterogeneity of factors that may influence implementation of PrEP in family planning clinics: A latent profile analysis. Implement Sci Commun. 2021;2(1):48. doi:10.1186/s43058-021-00148-3 33947472 PMC8097793

[34] Saberi P Berrean B Thomas S Gandhi M Scott H . A simple Pre-exposure prophylaxis (PrEP) optimization intervention for health care providers prescribing PrEP: Pilot study. JMIR Form Res. 2018;2(1):e2. doi:10.2196/formative.8623 PMC6325636 30637375

[35] Shrestha I Ming K Jimenez V , et al. Lessons learned from an HIV Pre-exposure prophylaxis coordination program in San Francisco primary care clinics. AIDS Res Hum Retroviruses. 2022;38(8):611‐614. doi:10.1089/aid.2022.0013 35592996 PMC9419977

[36] OHESI. Current Trends for HIV in Ontario. OHESI/OHTN; 2023, https://www.ohesi.ca

[37] Candler E Naeem Khan M Gratrix J , et al. Retrospective audit of a convenience cohort of individuals on HIV pre-exposure prophylaxis in Alberta, Canada. Off J Assoc Med Microbiol Infect Dis Can. 2022;7(4):350‐363. 10.3138/jammi-2022-0016 PMC10312220 37397818

[38] OHTN. HIV Services in Rural and Remote Communities. OHTN; 2013, https://www.ohtn.on.ca/wp-content/uploads/2016/12/RR73-2013-Rural-ASO.pdf

[39] Kamitani E Mizuno Y Koenig LJ . Strategies to eliminate inequity in PrEP services in the US south and rural communities. J Assoc Nurses AIDS Care JANAC. 2024;35(2):153‐160. doi:10.1097/JNC.0000000000000437 37963267 PMC11090982

[40] Bonett S Li Q Sweeney A Gaither-Hardy D Safa H . Telehealth models for PrEP delivery: A systematic review of acceptability, implementation, and impact on the PrEP care Continuum in the United States. AIDS Behav. 2024;28(9):2875‐2886. doi:10.1007/s10461-024-04366-3 38856846 PMC11390827

[41] Kamitani E Higa DH Crepaz N Wichser M Mullins MM The US . Centers for Disease Control and Prevention’s Prevention Research Synthesis Project. Identifying best practices for increasing HIV pre-exposure prophylaxis (PrEP) use and persistence in the United States: A systematic review. AIDS Behav. 2024;28(7):2340–2349. doi:https://doi.org/10.1007/s10461-024-04332-z 38743381 10.1007/s10461-024-04332-z PMC11199112

[42] Butts SA Young B Blackmon J Doblecki-Lewis S . Addressing disparities in pre-exposure prophylaxis (PrEP) access: Implementing a community-centered mobile PrEP program in south Florida. BMC Health Serv Res. 2023;23(1):1311. doi:10.1186/s12913-023-10277-1 38012701 PMC10683210

[43] Mantell JE Bauman LJ Bonett S , et al. Innovation in providing equitable pre-exposure prophylaxis services in the United States: Expanding access in nontraditional settings. JAIDS J Acquir Immune Defic Syndr. 2025;98(5S):e156. Accessed June 12, 2025. https://journals.lww.com/jaids/fulltext/2025/04151/innovation_in_providing_equitable_pre_exposure.19.aspx. doi:10.1097/QAI.0000000000003610 PMC12987501 40163068

[44] Briggs J Chiosi J Papineni S Murray M . Strategies to improve PrEP adherence in people who use drugs: A systematic review. AIDS Behav. 2026;30(2):403‐417. doi:10.1007/s10461-025-04876-8 41016995 PMC12560827

[45] Bazzi AR Shaw LC Biello KB Vahey S Brody JK . Patient and provider perspectives on a novel, low-threshold HIV PrEP program for people who inject drugs experiencing homelessness. J Gen Intern Med. 2023;38(4):913‐921. doi:10.1007/s11606-022-07672-5 PMC9132566 35614171

[46] Reback CJ Clark KA Rünger D Fehrenbacher AE . A promising PrEP navigation intervention for transgender women and men who have sex with men experiencing multiple syndemic health disparities. J Community Health. 2019;44(6):1193–1203. doi:https://doi.org/10.1007/s10900-019-00705-x 31317438 10.1007/s10900-019-00705-x PMC6859945

[47] Rhodes SD Alonzo J Mann-Jackson L , et al. A peer navigation intervention to prevent HIV among mixed immigrant status Latinx GBMSM and transgender women in the United States: Outcomes, perspectives and implications for PrEP uptake. Health Educ Res. 2020;35(3):165‐178. doi:10.1093/her/cyaa010 32441760 PMC7243724

[48] Martinez-Cajas JL Cofie N Kuforiji O , et al. Assessing the educational impact of a new HIV PrEP training module among primary care providers in southeast Ontario: Results from immediate and 3-months post-training evaluation surveys. AIDS Care. 2025:1‐14. doi:10.1080/09540121.2025.2594610 41330397

[49] Bhatia R Modali L Lowther M , et al. Outcomes of preexposure prophylaxis referrals from public STI clinics and implications for the preexposure prophylaxis Continuum. Sex Transm Dis. 2018;45(1):50. doi:10.1097/OLQ.0000000000000690 28876282

[50] Bruxvoort KJ Schumacher CM Towner W , et al. Referral linkage to preexposure prophylaxis care and persistence on preexposure prophylaxis in an integrated health care system. JAIDS J Acquir Immune Defic Syndr. 2021;87(3):918. doi:10.1097/QAI.0000000000002668 33633035

[51] Delivery models of pharmacy-led HIV pre-exposure prophylaxis (PrEP)—The Ontario HIV Treatment Network. January 9, 2025. Accessed February 7, 2025. https://www.ohtn.on.ca/rapid-response-delivery-models-of-pharmacy-led-hiv-pre-exposure-prophylaxis-prep/

