# Supplementary material for: Determinants of Adoption, Implementation, Reach, and Sustainability of PrEP Services in a Sexual Health Clinic in Canada: A Qualitative Analysis Using CFIR and REAIM
Source: J Int Assoc Provid AIDS Care. 2026 Jun 11;25:23259582261458932. doi: 10.1177/23259582261458932 (PMC13260786; doi:10.1177/23259582261458932)
Supplement: sj-docx-1-jia-10.1177_23259582261458932 - Supplemental material for Determinants of Adoption, Implementation, Reach, and Sustainability of PrEP Services in a Sexual Health Clinic in Canada: A Qualitative Analysis Using CFIR and REAIM [file sj-docx-1-jia-10.1177_23259582261458932.docx]

| **SRQR item** | **How it is addressed in this study** | **Page** |
| --- | --- | --- |
| **1. Title** | Indicate that this is a qualitative or mixed-methods implementation evaluation of a PrEP clinic, using RE-AIM, CFIR, and a health equity lens. | 1 |
| **2. Abstract** | Summarize background, objective, design, setting, participants, frameworks, data sources, main findings, and implications for PrEP implementation. | 1 and 2 |
| **3. Problem formulation** | Describe inequities in PrEP access and the need to understand how sexual health clinic models can support equitable PrEP delivery. | Page 4 and 5 |
| **4. Purpose / research questions** | State that the study examined adoption, implementation, reach, effectiveness, and maintenance of a PrEP clinic, and how CFIR/HEIF determinants shaped these outcomes. | Page 5 and 6 |
| **5. Qualitative approach and research paradigm** | Describe the qualitative approach, for example, framework-informed qualitative evaluation using RE-AIM, CFIR, and HEIF. | Page 7 and 8 |
| **6. Researcher characteristics and reflexivity** | Report researchers’ roles, disciplinary backgrounds, relationship to the clinic, and how reflexivity was addressed during data collection and analysis. | Page 13 |
| **7. Context** | Describe the mid-sized Ontario public health unit, sexual health clinic setting, regional PrEP access context, and policy/funding environment. | Page 6 |
| **8. Sampling strategy** | Explain how staff, leadership, clients, and other participants were recruited, including eligibility criteria and rationale for purposive or convenience sampling. | Page 10 and 11 |
| **9. Ethical issues** | Report ethics approval, informed consent, confidentiality procedures, data storage, and protections for participants discussing sexual health care. | Page 2 |
| **10. Data collection methods** | Describe interviews, process mapping, and present interview guides | Supplementary material and pages 10 and 11 |
| **11. Data collection instruments and technologies** | Describe interview guides, process mapping materials, recording/transcription methods, and software used for data management or coding. | Supplementary material |
| **12. Units of study** | Report the number and type of participants, such as staff, leadership, clients, and/or external providers, and describe relevant characteristics. | Page 13 |
| **13. Data processing** | Explain transcription, de-identification, data cleaning, and preparation of qualitative | Page 12 and 13 |
| **14. Data analysis** | Describe coding approach, use of RE-AIM/CFIR/HEIF frameworks, deductive and/or inductive coding, team coding, coding comparison, and development of themes. | Page 12 and 13 |
| **15. Techniques to enhance trustworthiness** | Report triangulation across data sources, use of multiple coders, analytic meetings, audit trail, reflexive memoing, and use of participant quotations. | Pages 12 and 13, and discussion |
| **16. Synthesis and interpretation** | Present findings by RE-AIM domains and interpret them using CFIR/HEIF determinants and health equity considerations. | Results section organize by RE-AIM and determinants |
| **17. Links to empirical data** | Include representative quotations from staff and clients, and link these to themes such as adoption, implementation fidelity, reach, effectiveness, and maintenance. | Results section |
| **18. Integration with prior work / theory** | Discuss how findings align with existing literature on PrEP implementation, telehealth, public health clinic models, equity-oriented care, and implementation frameworks. | Discussion |
| **19. Limitations** | Address possible selection bias, under-representation of disengaged clients or structurally marginalized groups, single-clinic context, and primarily qualitative design. | Page 39 and 40 |
| **20. Conflicts of interest** | Report any conflicts of interest or state that none were declared. | Page 1 |
| **21. Funding** | Report funding sources and describe the role of funders, if any, in study design, data collection, analysis, interpretation, or manuscript preparation. | Page 2 |
