# Supplementary material for: Determinants of Adoption, Implementation, Reach, and Sustainability of PrEP Services in a Sexual Health Clinic in Canada: A Qualitative Analysis Using CFIR and REAIM
Source: J Int Assoc Provid AIDS Care. 2026 Jun 11;25:23259582261458932. doi: 10.1177/23259582261458932 (PMC13260786; doi:10.1177/23259582261458932)
Supplement: sj-docx-2-jia-10.1177_23259582261458932 - Supplemental material for Determinants of Adoption, Implementation, Reach, and Sustainability of PrEP Services in a Sexual Health Clinic in Canada: A Qualitative Analysis Using CFIR and REAIM [file sj-docx-2-jia-10.1177_23259582261458932.docx]

**Supplementary material**

**Table 1 Implementation outcomes used in addition to those proposed by RE-AIM**

| **Service Outcome** | **Definition (Proctor et al.)*** | **Application to PrEP Services** |
| --- | --- | --- |
| **Acceptability- (**staff and clients**)** | The perception among stakeholders that a service is agreeable, palatable, or satisfactory. | Staff and clients’ satisfaction with PrEP services, including counseling approach, clinical interactions, follow-up frequency, and overall experience of PrEP care.  Staff perceptions of workload and comfort delivering PrEP |
| **Appropriateness**  **(**staff and clients**)** | The perceived fit, relevance, or compatibility of a service for a given setting or population. | Staff and clients’ perceptions of whether PrEP services are relevant and suitable for local populations (e.g., gbMSM, trans women, people who use drugs, immigrants) and service contexts.  Alignment of PrEP services with clinic mandate and client needs |
| **Fidelity**  **(**staff and clients**)** | The degree to which a service is delivered as intended by its developers. | The extent to which PrEP services are delivered according to clinical guidelines (e.g., eligibility assessment, monitoring schedules, adherence counseling). |
| **Cost**  **(**staff and clients**)** | The costs associated with implementing and delivering PrEP services | Laboratory and medication costs. Costs related to training, navigation, and administration staff time required for PrEP initiation and follow-up |

* Proctor E, Silmere H, Raghavan R, Hovmand P, Aarons G, Bunger A, Griffey R, Hensley M. Outcomes for implementation research: conceptual distinctions, measurement challenges, and research agenda. Adm Policy Ment Health. 2011 Mar;38(2):65-76. doi: 10.1007/s10488-010-0319-7. PMID: 20957426; PMCID: PMC3068522.

**Table 2. Service outcomes used in addition to those proposed in the effectiveness domain of RE-AIM**

| **Service Outcomes** | **Definitions (Proctor et al)** | **Application to PrEP services** |
| --- | --- | --- |
| **Efficiency** | Avoiding waste, including waste of equipment, supplies, ideas, and energy | Streamlined laboratory monitoring schedules aligned with guidelines  Use of telehealth or hybrid visits to reduce unnecessary in-person appointments  Low rates of duplicated testing or missed appointments |
| **Safety** | Avoiding harm to patients from the care that is intended to help them | Timely identification and management of PrEP side effects  Safe prescribing practices and medication reconciliation  Clear protocols for HIV testing to avoid PrEP initiation during acute infection |
| **Equity** | Providing care that does not vary in quality because of personal characteristics such as gender, ethnicity, geographic location, and socioeconomic status | Availability of PrEP navigation, language support, and culturally appropriate counseling;  Reduction of financial barriers through drug coverage or subsidy programs |
| **Patient centered** | Providing care that is respectful of and responsive to individual patient preferences,  needs, and values and ensuring that patient values guide all clinical decisions | Flexible follow-up schedules based on client needs  Non-judgmental, trauma-informed counseling approaches  Client-reported satisfaction and perceived respect in care |
| **Timeliness** | Reducing waits and sometimes harmful delays for both those who receive and those who give care | Same-day or rapid PrEP starts when clinically appropriate; T  imely laboratory result turnaround and follow-up communication  Reduced wait times for appointments and prescription refills |

* Proctor E, Silmere H, Raghavan R, Hovmand P, Aarons G, Bunger A, Griffey R, Hensley M. Outcomes for implementation research: conceptual distinctions, measurement challenges, and research agenda. Adm Policy Ment Health. 2011 Mar;38(2):65-76. doi: 10.1007/s10488-010-0319-7. PMID: 20957426; PMCID: PMC3068522.

**Table 3. CFIR definitions used to code the interviews to identify determinants of each RE-AIM**

| **Domain** | **Determinant** | **Definition (PrEP-focused; Staff and Clients)** |
| --- | --- | --- |
| **Intervention Characteristics** | Intervention source  (not found) | Staff and clients’ perceptions of whether PrEP services are externally developed (e.g., public health– or guideline-driven) or internally developed and adapted within the organization. |
|  | Evidence strength and quality | Staff and clients’ perceptions of the quality, credibility, and relevance of evidence supporting PrEP effectiveness. |
|  | Relative advantage | Staff and clients’ perceptions of the advantages of PrEP compared with alternative HIV prevention strategies. |
|  | Adaptability | The degree to which PrEP delivery can be tailored to meet local client needs and organizational contexts. |
|  | Trialability | The ability to pilot PrEP services on a small scale and modify or discontinue delivery if needed. |
|  | Complexity | Perceived difficulty of implementing PrEP, including clinical workflows, monitoring, and follow-up requirements. |
|  | Design quality and packaging | Perceived quality of how PrEP services, clinical protocols (medical directives), and educational materials are presented and integrated. |
|  | Cost | Direct and indirect costs associated with PrEP delivery, including medication, laboratory testing, staffing, and opportunity costs. |
| **Outer Setting** | **Critical incidents** | External events (e.g., local HIV outbreaks, changes in drug coverage, service disruptions, COVID-19) that influence the prioritization, adaptation, or continuity of PrEP services. |
|  | **Local attitudes** | Prevailing community attitudes, beliefs, and norms related to PrEP, HIV, sexuality, substance use, and key populations, as perceived by staff and clients. |
|  | **Local conditions** | Social, economic, geographic, and structural conditions (e.g., rurality, poverty, stigma, migration, access to healthcare) that shape access to and sustained engagement with PrEP. |
|  | **Policies and laws** | Provincial or national policies, clinical guidelines, regulations, and legal frameworks that affect PrEP eligibility, prescribing, monitoring, and service delivery. |
|  | **Networking / partnerships** | The extent and quality of organizational connections with external partners (e.g., primary care, pharmacies, AIDS service organizations, harm reduction programs) that support PrEP referral, navigation, and continuity of care. |
|  | **Financing** | External funding and coverage mechanisms (e.g., public drug plans, insurance, subsidies, grants) that influence the affordability, scalability, and sustainability of PrEP services. |
|  | **External pressure** | Explicit or implicit pressure from public health authorities, funders, professional networks, or peer organizations to implement or expand PrEP services. |
| **Inner Setting** | Structural characteristics | Organizational features such as size, maturity, staffing model, and service scope that shape PrEP delivery capacity. |
|  | Networks and communication | Quality of formal and informal communication among staff involved in PrEP delivery. |
|  | Culture | Organizational norms and values related to sexual health, harm reduction, and equity. |
|  | Implementation climate | The extent to which PrEP delivery is expected, supported, and rewarded. |
|  | Tension for change | Staff and clients’ perceptions that existing HIV prevention approaches are insufficient, creating a need for PrEP. |
|  | Compatibility | The degree of fit between PrEP services and existing workflows and systems. |
|  | Relative priority | The perceived importance of PrEP relative to other organizational initiatives. |
|  | Learning climate | An environment characterized by openness, reflection, and support for learning around PrEP delivery. |
|  | Readiness for implementation | Observable indicators of organizational commitment to implementing PrEP. |
|  | Leadership engagement | The commitment and involvement of leaders in supporting PrEP implementation. |
|  | Available resources | Availability of time, funding, staffing, and infrastructure to support PrEP delivery. |
|  | Access to knowledge and information | Ease of access to PrEP-related training, protocols, and informational resources. |
| **Characteristics of Individuals** | Knowledge and beliefs about PrEP | Staff and clients’ understanding of PrEP and beliefs about its effectiveness and appropriateness. |
|  | Self-efficacy | Confidence of staff in delivering PrEP and of clients in initiating and adhering to PrEP. |
|  | Compatibility of PrEP with personal values | The degree to which staff identify with and feel committed to the organization’s mission. |
| **Process** | Engaging | Strategies to attract and involve staff and clients in PrEP delivery. |
|  | Opinion leaders | Strategies to involve influential individuals who shape attitudes toward PrEP. |
|  | Planning | Delineation of strategies to imporve the delivery of services. |
|  | Executing | Carrying out PrEP implementation activities as intended. |
|  | Reflecting and evaluating | Ongoing assessment of PrEP implementation processes and outcomes. |

**Table 4. Interview guide for process mapping**

| **Section** | **Question** | **Purpose / Focus** |
| --- | --- | --- |
| **Role in the PrEP Process** | What role-specific steps (Nurse, DC, Physician, Manager) are you involved in within PrEP services for clients? | Identify responsibilities by professional role. |
|  | What does this process look like for you? | Understand individual workflow experience. |
|  | How are you involved? What specific tasks do you perform? | Clarify concrete duties and activities. |
|  | Is there anything you would like to share about how your role affects other team members’ roles in the process? | Explore team dynamics and interdependencies. |
| **Process Efficiency and Functioning** | What is helpful about the current process? | Identify facilitators and strengths. |
|  | What is challenging or difficult? | Identify barriers and bottlenecks. |
|  | How much time do you spend on the entire process with clients? | Assess workload and time allocation. |
|  | Do you think this time is well spent? Or could something help make your time more effectively used? | Explore perceptions of efficiency and improvement opportunities. |
| **Resources** | What resources do you require to carry out this process? | Identify necessary supports and tools. |
|  | Are additional resources needed to improve the process? | Identify gaps and resource needs. |
| **Client Support** | How do you support clients throughout the process? | Understand patient engagement and support strategies. |
|  | What resources do you provide to clients? | Identify materials and supports offered to clients. |
| **Process Improvement** | Is there a desire within the team to expand or improve the process? | Assess organizational readiness for change. |
|  | Do you have any suggestions, changes, or revisions to improve the process? | Gather recommendations for optimization. |

**Table 5. Interview guide to clinic staff using CFIR and RE-AIM**

| **Section** | **Question** | **Purpose / Focus** |
| --- | --- | --- |
| **Appropriateness** |  |  |
| Perceived Need | Is there a strong need to continue providing PrEP services in your organization? | Assess perceived necessity of the service. |
|  | Do you think others in the organization see a need for PrEP services? | Explore shared perceptions of need. |
| Organizational Fit | To what extent has the implementation of the PrEP clinic aligned with organizational goals and priorities? | Examine strategic alignment. |
|  | To what extent is the PrEP clinic integrated into organizational operations? | Assess integration into routine practice. |
| Cultural Fit | To what extent were clients’ cultures and/or values considered when designing the services? Please describe. | Assess cultural responsiveness. |
|  | In what ways is PHU organizational culture different from or similar to other settings (healthcare providers, PHUs, community partners, HIV partners)? | Compare organizational context and compatibility. |
| **Acceptability** |  |  |
| Client Acceptability | What factors are important to ensure acceptance of PrEP services among clients? | Identify determinants of client acceptance. |
| Staff Acceptability | What factors were considered important to ensure staff acceptance (nurses, DC, physicians)? | Assess staff engagement and perceptions. |
|  | What are staff perceptions of PrEP services (positive/negative experiences)? | Explore implementation climate. |
| External Stakeholders | What would be needed for other healthcare providers to find the PrEP clinic meaningful? | Identify external adoption facilitators. |
| **Adoption** |  |  |
| Adoption Process | How would you describe the process of adopting PrEP services in your organization? | Document decision-making process. |
|  | Who was engaged in the decision to implement PrEP services? | Identify key stakeholders. |
|  | Has any population been missed during adoption? | Assess equity in adoption. |
| Staffing & Engagement | Which providers and staff were engaged? Have there been changes? | Examine staffing evolution. |
|  | What staffing variables need to be considered? What expertise and motivation are required? | Identify workforce requirements. |
| External Partnerships | What external healthcare partners supported development? | Identify collaborative influences. |
|  | What challenges exist for other providers adopting PrEP? | Explore external barriers. |
|  | What recommendations do you have for training external providers? | Inform scale-up strategies. |
| **Planning** |  |  |
| Training & Preparedness | How well equipped did you feel to deliver PrEP services based on your training? | Assess perceived readiness. |
|  | What are your perceptions of the training provided? | Evaluate training quality. |
| **Implementation** |  |  |
| Implementation Process | Were PrEP services implemented according to plan? Were modifications needed? Why? | Assess fidelity and adaptation. |
|  | Describe the process for modifying the plan and its impact. | Understand adaptation mechanisms. |
| Infrastructure & Approvals | What infrastructure changes and approvals were required? | Identify structural adjustments. |
| Monitoring & Evaluation | How do you report adaptations? How do you monitor fidelity? | Assess quality assurance systems. |
|  | How were decisions made about tracking process and outcomes? | Understand evaluation strategy. |
| Client Pathways | What mechanisms are in place to help clients access PrEP? | Map support systems. |
|  | What happens if someone declines PrEP? | Explore alternative pathways. |
|  | What influences clients’ decisions about PrEP? | Identify decision determinants. |
| Process Changes | Have there been recent process changes? | Identify iterative improvements. |
|  | Have new materials or roles been introduced? | Examine innovation. |
| Staff Experience | What were your initial thoughts about offering PrEP? | Capture implementation climate. |
|  | Was there resistance? How smooth was the transition? | Assess organizational change dynamics. |
| Improvement | If you could change one thing about the PrEP clinic, what would it be and why? | Identify improvement priorities. |
| Cost Considerations | What costs were considered during implementation? | Assess economic planning. |
|  | Were there overlooked costs? | Identify planning gaps. |
|  | How do costs impact adoption or maintenance? | Evaluate sustainability risks. |
|  | How does PrEP cost compare to other interventions offered? | Contextualize economic burden. |
| **Reach** |  |  |
| Population Served | What populations are served? Are any missing? | Assess representativeness. |
| Recruitment & Outreach | What advertising methods are used? | Identify outreach strategies. |
|  | How well does recruitment work? | Evaluate recruitment effectiveness. |
|  | Did recruitment meet expectations? | Assess performance. |
|  | What worked best? What could be improved? | Identify best practices. |
| Client Characteristics | What characteristics are common among clients starting PrEP? | Profile adopters. |
|  | Are some groups more open to PrEP? | Assess differential uptake. |
|  | What characteristics are common among those not participating? | Identify gaps. |
|  | Why do some clients decline? | Identify barriers. |
|  | Who is not being reached? | Examine inequities. |
|  | How do clients learn about the clinic? | Assess referral pathways. |
|  | How could reach be improved? | Identify equity strategies. |
| **Effectiveness** | Which clients benefit most? | Identify differential effectiveness. |
|  | What are the main outcomes of the service? | Clarify intended effects. |
|  | What are barriers to effectiveness? | Identify limiting factors. |
|  | How do you know the intervention is working? | Assess evidence use. |
|  | How effective is PrEP in improving sexual health? | Evaluate perceived impact. |
|  | How should outcomes be collected and presented? | Inform future evaluation design. |
|  | Is this intervention more helpful than previous processes? | Comparative effectiveness. |
| **Maintenance** |  |  |
| Sustainability Risks | Are there barriers to maintaining PrEP services? | Identify threats. |
|  | What concerns exist about continuing services? | Assess sustainability perception. |
|  | What resources are needed for continuation? | Identify sustainability supports. |
| Continuation Likelihood | What is the likelihood of continuing this intervention? | Assess sustainability commitment. |
| Future Capacity | What would be needed for staff to deliver this in the future? | Identify workforce development needs. |
|  | What is necessary to build skills and confidence? | Inform capacity building. |
|  | What long-term improvements do you foresee? | Identify strategic outlook. |

**Table 6. Interview guide with clients of the PrEP clinic**

| **Section** | **Question** | **Purpose / Focus** |
| --- | --- | --- |
| **Awareness** | Had you heard about PrEP before becoming a client of the PrEP clinic? | Assess baseline awareness. |
|  | Where did you first learn about PrEP? | Identify information sources and referral pathways. |
| **Adoption** | What motivated you to use PrEP? | Explore drivers of uptake. |
|  | What do you like about PrEP? | Identify perceived advantages. |
|  | Why did you think PrEP was a good fit for you? | Assess perceived personal relevance. |
| **Acceptability, implementation** |  |  |
| Facilitators | What made you interested in receiving services at the KFL&A Public Health PrEP clinic? | Identify initial engagement factors. |
| Barriers | Have you encountered any challenges or barriers while participating in the clinic? | Identify access or process barriers. |
| Overall Experience | Can you describe your experience with the PrEP services so far? | Capture general satisfaction and experience. |
| Access & Logistics | How easy or difficult was it to access PrEP services (e.g., obtaining lab work, prescriptions, filling prescriptions, scheduling appointments)? | Assess service accessibility and workflow efficiency. |
| Setting | What do you think of the location, setting, and appointment times? | Evaluate convenience and environmental factors. |
| Staff Experience | What do you think of the staff who delivered PrEP services? | Assess interpersonal and relational aspects of care. |
| **Effectiveness** |  |  |
| Goals | What was your goal in receiving PrEP services? Did you achieve it? | Assess alignment between expectations and outcomes. |
| Additional Benefits | Were there other benefits of using PrEP (e.g., physical, mental, or sexual health)? | Explore broader impacts. |
| Concerns | Do you have any concerns or reservations about using PrEP? | Identify ongoing hesitations or perceived risks. |
| Discontinuation (if applicable) | If you stopped using PrEP, what were your reasons? | Identify barriers to continuation. |
| **Fidelity** |  |  |
| Clinic Support | Did you receive sufficient information and support from healthcare providers or program staff? | Assess adequacy of support. |
| Additional Resources | Were there additional resources or support services within the clinic that were helpful? | Identify supportive elements. |
|  | Were there any resources outside the PrEP clinic that were useful to you? | Explore external supports. |
| **Maintenance** | Question | Purpose / Focus |
| Continuation | Would you consider continuing to use PrEP in the future? Why or why not? | Assess maintenance intentions. |
| Influencing Factors | What factors would influence your decision to start or stop using PrEP in the future? | Identify determinants of sustained use. |
|  | Are any of these factors related to how you receive PrEP at the KFL&A clinic? | Link service delivery to retention. |
| **Recommendations** |  |  |
| Service Improvement | What recommendations do you have for improving PrEP service delivery at KFL&A? | Identify actionable improvements. |
| Word of Mouth | Have you recommended the PrEP clinic to someone? Would you recommend it to a friend? | Assess satisfaction and advocacy. |
| Program Enhancement | Based on your experience, how could the clinic better meet clients’ needs? | Gather client-centered improvement ideas. |
| Outreach | What are the best ways to inform people about the program? | Inform recruitment strategies. |
| **Closing** | Is there anything else you would like to add about your experience with the PrEP clinic? | Provide opportunity for additional insights. |
